# NO-sensitive guanylyl cyclase discriminates pericyte-derived interstitial from intra-alveolar myofibroblasts in murine pulmonary fibrosis

**DOI:** 10.1186/s12931-023-02479-2

**Published:** 2023-06-22

**Authors:** Annemarie Aue, Nils Englert, Leon Harrer, Fabian Schwiering, Annika Gaab, Peter König, Ralf Adams, Achim Schmidtko, Andreas Friebe, Dieter Groneberg

**Affiliations:** 1 Physiologisches Institut, Julius-Maximilians-Universität Würzburg, Röntgenring 9, 97070 Würzburg, Germany; 2 Zentrum für Interdisziplinäre Schmerzmedizin, Klinik für Anästhesiologie Intensivmedizin, Notfallmedizin und Schmerztherapie, Universitätsklinikum Würzburg, 97080 Würzburg, Germany; 3 Institut für Anatomie, Zentrum für Medizinische Struktur- und Zellbiologie, Universität zu Lübeck, 23562 Lübeck, Germany; 4 Airway Research Center North (ARCN), German Center for Lung Research (DZL), Giessen, Germany; 5 Max-Planck-Institute for Molecular Biomedicine, Department of Tissue Morphogenesis, Faculty of Medicine, University of Münster, 48149 Münster, Germany; 6 Institut für Pharmakologie und Klinische Pharmazie, Goethe-Universität Frankfurt, Max-von-Laue-Str. 9, 60438 Frankfurt am Main, Germany

**Keywords:** Guanylyl cyclase, Myofibroblasts, Pericytes, Transgenic mouse, Fibrosis

## Abstract

**Background:**

The origin of αSMA-positive myofibroblasts, key players within organ fibrosis, is still not fully elucidated. Pericytes have been discussed as myofibroblast progenitors in several organs including the lung.

**Methods:**

Using tamoxifen-inducible PDGFRβ-tdTomato mice (PDGFRβ-CreER^T2^; R26tdTomato) lineage of lung pericytes was traced. To induce lung fibrosis, a single orotracheal dose of bleomycin was given. Lung tissue was investigated by immunofluorescence analyses, hydroxyproline collagen assay and RT-qPCR.

**Results:**

Lineage tracing combined with immunofluorescence for nitric oxide-sensitive guanylyl cyclase (NO-GC) as marker for PDGFRβ-positive pericytes allows differentiating two types of αSMA-expressing myofibroblasts in murine pulmonary fibrosis: (1) interstitial myofibroblasts that localize in the alveolar wall, derive from PDGFRβ^+^ pericytes, express NO-GC and produce collagen 1. (2) intra-alveolar myofibroblasts which do not derive from pericytes (but express PDGFRβ de novo after injury), are negative for NO-GC, have a large multipolar shape and appear to spread over several alveoli within the injured areas. Moreover, NO-GC expression is reduced during fibrosis, i.e., after pericyte-to-myofibroblast transition.

**Conclusion:**

In summary, αSMA/PDGFRβ-positive myofibroblasts should not be addressed as a homogeneous target cell type within pulmonary fibrosis.

**Supplementary Information:**

The online version contains supplementary material available at 10.1186/s12931-023-02479-2.

## Background

Lung fibrosis is a chronic disease characterized by alveolar epithelial injury, myofibroblast differentiation, deposition of extracellular matrix (ECM), dysregulated inflammation and formation of scar tissue [1, 2]. The underlying mechanisms are not fully understood, and effective therapies are scarce [3, 4]. Myofibroblasts act as key regulators of wound repair by producing extracellular matrix, cytokines and chemokines as well as growth factors. The origin of myofibroblasts is still being debated, but recent evidence suggests resident pericytes to be of major importance as myofibroblast progenitors in the lung [5–8].

The unequivocal identification of pericytes in various organs has been challenging and so far, unique markers have not been identified. In addition, pericytes may be morphologically diverse depending on organ and development/disease state [9]. Several proteins have been identified as relatively specific markers such as platelet-derived growth factor β (PDGFRβ), desmin, neural/glial antigen 2 (NG2), α smooth muscle actin (αSMA) or CD146. In previous studies, we have identified NO-sensitive guanylyl cyclase (NO-GC), the main receptor for NO, to be expressed in pericytes of various organs including the lung [10, 11].

In this study, we characterized NO-GC expression in pulmonary pericytes and in smooth muscle cells (SMC). In addition, we identified the enzyme as novel pericyte marker to monitor pericyte-to-myofibroblast transformation. Following bleomycin injury, lineage tracing shows two types of αSMA-expressing myofibroblasts that can be differentiated by NO-GC but not by PDGFRβ expression: pericyte-derived, NO-GC^+^ myofibroblasts were found in the alveolar interstitium whereas NO-GC^−^ myofibroblasts transiently occupied former alveolar cavities (termed ‘intra-alveolar’ myofibroblasts throughout this manuscript). In addition, NO-GC-expressing pericyte-derived myofibroblasts produce col1α1 during fibrosis. Based on their differential tissue distribution these myofibroblasts are likely to have individual functions and, thus, may constitute potential therapeutic targets to influence fibrotic processes**.**

## Methods

### Animals

Mice were housed in standard mouse cages (267 × 207 × 140 mm; maximally three animals/cage) with woodchip bedding material and under conventional laboratory conditions (constant room temperature (22 °C), humidity level (55%), a 12-h light/12-h dark cycle (lights on at 6 am), and standard rodent diet and water available ad libitum. A total of 76 animals of both sexes was used.

Wildtype (WT) mice with a C57BL/6 J genetic background were used for non-lineage tracing experiments. For lineage tracing studies, PDGFRβ-tdTomato reporter mice expressing the fluorescent dye tdTomato under control of the PDGFRβ promotor were obtained by crossing PDGFRβ-CreER^T2^ mice (JAX #029684; genetic background: C57Bl6/129SV) with a tdTomato reporter line (Ai14; JAX #007914; genetic background: C57Bl6).

### Tamoxifen injection

PDGFRβ-tdTomato reporter mice aged 6–8 weeks were injected with tamoxifen (dissolved in Miglyol 812; 1 mg i.p.) on 5 consecutive days followed by bleomycin treatment 50 days later.

### Bleomycin administration

Bleomycin sulfate was prepared in 0.9% sodium chloride solution given orotracheally (2 U/kg body weight) to isoflurane-anaesthetized mice. Initial tests showed no difference between untreated control mice and control mice receiving the same volume of 0.9% sodium chloride solution; therefore, untreated mice served as controls. Bleomycin-induced lung injury was assessed after 21 days, fibrosis resolution after 56 days. Specific treatment regimens are indicated in the respective figures/figure legends.

### Immunofluorescence analysis

After euthanasia, lungs were perfused with 0.9% sodium chloride solution and 4% paraformaldehyde (PFA) through the right ventricle. Using a 20-gauge needle through a small incision into the trachea, lungs were inflated to 24 cm H_2_O pressure with 4% PFA in 0.1 M phosphate buffer, pH 7.4. Inflated lungs were removed and fixed with 4% PFA for 20 min. The tissue was incubated overnight in 20% sucrose in phosphate buffer and subsequently snap frozen. Cryosections (10 µm) were cut, air-dried, permeabilized and incubated overnight with the following primary antibodies: homemade antibody against the β_1_ subunit of NO-GC (~ 360 N-terminal amino acids fused to glutathione-S-transferase, specificity frequently shown with mice lacking NO-GC, e.g., [12]) raised in rabbit (1:800), goat anti-desmin antibody (ABIN334386; 1:200; antibodies-online GmbH, Aachen, Germany), goat anti-PDGFRβ antibody (AF1042; 1:200; R&D Systems, Minneapolis, USA), rabbit anti-TTF1 antibody (ab227652; 1:100; Abcam, Cambridge, UK), rat anti-NG2 antibody (MAB6689; 1:100; R&D Systems, Minneapolis, USA), rat anti-CD146 antibody (MAB7718; 1:200; R&D Systems, Minneapolis, USA), rabbit anti-collagen type 1α1 (col1α1) antibody (ab34710; 1:500; Abcam, Cambridge, UK), rabbit anti-laminin 1 antibody (BP8037; 1:200; OriGene, Herford, Germany), rat anti-CD31 antibody (cat. No. 550274; 1:200; BD Biosciences, Heidelberg, Germany), mouse anti-αSMA FITC-conjugated antibody (F3777; 1:500; clone 1A4, Sigma-Aldrich, München, Germany), rabbit anti-αSMA antibody (ab5694; 1:200; Abcam, Cambridge, UK) and rat anti-receptor for advanced glycation endproducts (RAGE) antibody (ABIN360934; 1:100; Antibodies-online Inc., Atlanta, USA). Secondary antibodies were incubated in antibody diluent either alone or in combination for one hour: Rabbit antibodies were detected by a donkey anti-rabbit Alexa 555-IgG antibody (A-31572; 1:500; Invitrogen, Darmstadt, Germany), rat antibodies were detected by a donkey anti-rat Alexa-488- (A-21208; 1:500; Invitrogen, Darmstadt, Germany) or Alexa-647-conjugated IgG antibody (A78947; 1:500; Invitrogen, Darmstadt, Germany) and goat antibodies were detected by a donkey anti-goat Alexa 647-conjugated IgG antibody (A-21447; 1:500; Invitrogen, Darmstadt, Germany). Samples were stained with DAPI (A4099; 1:1000; Applichem, Heidelberg, Germany) for 7 min. The sections were mounted in Mowiol and were evaluated using a confocal microscope (Leica TCS SP8). Signal intensity was displayed using pseudocolor image processing with Fiji. Pseudocolors range from yellow (high signal intensity) to purple (low signal intensity). As an alternative for bright field (BF) microscopy, we used a differential interference contrast polarizing filter (DIC/pol). Polarized light was used for visualization of tissue.

### Quantification of 20 × immunofluorescence images

In order to quantify area of αSMA, PDGFRβ and NO-GC immunostaining and to count DAPI- or tdTomato^+^ cell nuclei, representative 20 × images from different animals (n = 9 images from N = 3 animals) were captured under identical settings and quantified using macro scripts for Fiji. One quarter (290 µm × 290 µm) of a whole 20 × image (580 µm × 580 µm) was analyzed. Regions of interest (ROI) were chosen without SMC of blood vessels and bronchi to selectively quantify pericytic (PDGFRβ, NO-GC) or myofibroblastic (αSMA, PDGFRβ) expression. DAPI^+^ or tdTomato^+^ cell nuclei were counted using macro scripts for Fiji.

### Cell counting of 63 × immunofluorescence images

Cells of total 63 × images (N = 3 animals with n = 3 images per animal) were counted manually. Before images were captured, ROI were chosen for best possible pericyte/myofibroblast analysis. tdTomato^+^ cells were counted based on cell nuclei co-expressing DAPI and tdTomato. Cell somata with DAPI^+^ nuclei, which were positive for αSMA-, NO-GC- or PDGFRβ-immunostaining, were counted to obtain pericyte/myofibroblast numbers. Macrophages and SMCs (if unavoidable) were excluded from counting.

### Hydroxyproline assay

Mice were killed by cervical dislocation and lungs were rinsed 3 times with 0.9% sodium chloride solution. The left pulmonary lobe was excised and dried to determine lung dry weight. Subsequently, dried tissue was hydrolyzed in 6 M HCl (100 µl per 1 mg) for 18–24 h at 115 °C and then centrifuged for 2 min at 9.500×*g*. The supernatants were dried in a Speed-Vac centrifuge and used for measurements of collagen in a hydroxyproline assay. The pellets were resuspended and diluted with water. Samples and standards were generated in triplicates and duplicates, respectively. Samples and standards were oxygenated with chloramine T for 20 min at room temperature. Ehrlich’s reagent was added, and the solutions were heated at 60 °C for 15 min. Then, the absorbance was determined spectrophotometrically at 560 nm and the hydroxyproline amount was calculated. The weights of the dried tissues and body weights were used for standardization.

### RT-qPCR

After euthanasia, lungs were perfused with 0.9% sodium chloride in DEPC water. The tissue was immediately snap frozen in liquid nitrogen and stored at − 20 °C. Using QIAshredder and Qiagen Rneasy® Mini Kit, total RNA was isolated from 15 mg lung tissue (right lung). RNA concentration and purity were assessed by a microvolume spectrophotometer (SimpliNano Spectrophotometer, Biochrom). cDNA was synthesized using 50 ng/µl of total RNA according to the High-Capacity cDNA Reverse Transcription Kit by Thermo Fisher Scientific. qPCR was performed with the CFX96 Real-Time PCR Detection System, Bio-Rad, using total cDNA as a template, specific primers for NO-GC and hypoxanthine guanine phosphoribosyltransferase (HPRT; as housekeeping gene) and the SsoFast EvaGreen® Supermix by Bio-Rad. The reaction procedure was as following: 98 °C (30 s); 40 cycles of 98 °C (5 s) and 65 °C (5 s); followed by a melt curve analysis: 65–95°C (in 0,5 °C inc.) for 5 s/step.

PrimerBlast by NCBI was used to design primers (Table 1) based on the mRNA sequence of Mus musculus. All primers were produced by Sigma-Aldrich, München.

**Table 1:** qPCR primers used

Gene	Primer	Sequence (5’-3’)
HPRT	Forward	GGTTAAGCAGTACAGCCCCA
	Reverse	TCCAACACTTCGAGAGGTCC
NO-GCβ_1_	Forward	TCTGCCAGGAGTCTGGCTAT
	Reverse	TAAATGGTGGCGAGGTGGTC

### Materials

Chloramine T trihydrate, trans-4-Hydroxy-L-proline, 4-(dimethylamino) benzaldehyde and tamoxifen and other standard chemicals were purchased from Sigma (Taufkirchen, Germany).

### Statistics

Data are expressed as mean ± SEM. For calculation of statistical tests, GraphPadPrism 9.0 for Windows was used. Two independent groups were compared by unpaired, two-sided T-test. One-way ANOVA followed by Tukey post-hoc test was used to compare multiple groups of one genotype.

## Results

### Immunofluorescence analysis of NO-GC expression in the lung

**Fig. 1:**
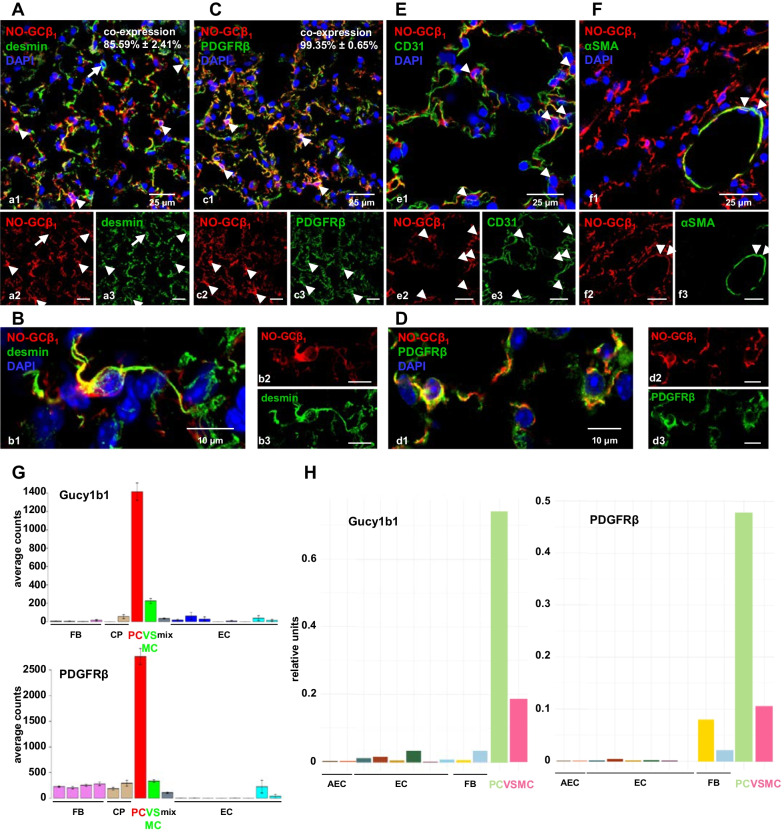
NO-GC expression in the murine lung. Lungs from WT mice (**A**–**F**) were stained with antibodies against the β_1_ subunit of NO-GC (NO-GCβ_1_) and desmin, PDGFRβ, CD31 or αSMA, respectively. Arrowheads in the merged images indicate cells which co-express NO-GC (red) with two established pericyte markers desmin (85.59% ± 2.41%; green, **A**; enlargement in **B**; desmin^+^/NO-GC^−^ cell indicated by arrow) and PDGFRβ (99.35% ± 0.65%; green, **C**; enlargement in **D**). **E** Signals for NO-GCβ_1_ (red) and CD31 (green) as marker for endothelial cells show close proximity but both markers are not co-expressed by the respective cells (arrowheads indicate NO-GC^+^/CD31^−^ cells which are in direct neighborhood to NO-GC^−^/CD31^+^ endothelial cells). **F** Co-expression of αSMA (green) and NO-GCβ_1_ (red) in blood vessels of the healthy lung indicates NO-GC expression in vascular smooth muscle cells (arrowheads). DAPI was used to stain nuclei (blue). For the quantitative analyses of the co-expression, a total of 773 (desmin) or 775 (PDGFRβ) DAPI^+^ cells were counted from n = 9 images (63 × magnification) from N = 3 animals. Single channels are shown in **a2**–**f2** and **a3**–**f3**. **G**, **H** Pericyte-specific scRNA expression of NO-GCβ_1_ subunit (Gucy1b1) and PDGFRβ in adult murine lung (http://betsholtzlab.org/VascularSingleCells/database.html; [18], and www.LungEndothelialCellAtlas.com; [19]). *AEC* alveolar epithelial cells, *FB* vascular fibroblast-like cells, *CP* cartilage perichondrium, *PC* pericytes, *VSMC* vascular smooth muscle cells, *EC* endothelial cells

We first used immunofluorescence to determine the cell type(s) that express NO-GC in murine lung. Lung tissue of WT mice was double stained with antibodies against NO-GCβ_1_ (the common subunit of both NO-GC isoforms, i.e., NO-GC1 and NO-GC2) and two pericyte markers, desmin and PDGFRβ. In addition, antibodies against CD31 and αSMA were used as established markers for endothelial cells and SMC, respectively. Figure 1A shows that desmin and NO-GC are co-expressed to a very high degree (85.59% ± 2.41%). Moreover, virtually all cells expressing PDGFRβ are immunoreactive for NO-GC (99.35% ± 0.65%; Fig. 1C). Desmin^+^/NO-GC^+^ (Fig. 1B) and PDGFRβ^+^/NO-GC^+^ cells (Fig. 1D) are multipolar and extend cytoplasmic processes. To verify that NO-GC is expressed in pericytes, two additional canonical pericyte markers, NG2 and CD146, were selected for triple stainings with antibodies against NO-GCβ_1_ and PDGFRβ, respectively (see Additional file 1: Fig. S1A–F). NG2- and CD146-positive cells co-express NO-GC (99.15% ± 0.85%; see Additional file 1: Fig. S1A, and 99.23% ± 0.74%; Additional file 1: Fig. S1D, respectively); these double-positive cells also express PDGFRβ (see Additional file 1: Fig. S1C and F). NG2^+^/NO-GC^+^ and CD146^+^/NO-GC^+^ cells are also multipolar and show fine extensions (compare Fig. 1B and D and Additional file 1: Fig. S1B and E). Thus, the combination of NO-GCβ_1_ and PDGFRβ labels pericytes in murine lung parenchyma. Given the shape, localization and marker expression, our data suggest that pericytes are the major NO-GC-expressing cell type in the murine lung.

NO-GC-expressing pericytes were found in close proximity to CD31-positive endothelial cells (arrowheads in Fig. 1E). Endothelial cells do not express NO-GC in murine lung; however, a small degree of the apparent 'co-stain' (yellow) is visible between pericytes and EC. This can be explained by the fact that pericytes are embedded within the basement membrane of microvessels, which is formed by both pericytes and endothelial cells, and by the delicate extensions with which pericytes wrap around endothelial cells ([13]; see Additional file 1: Fig. S1G–J). However, NO-GC was also found to be co-expressed with αSMA in blood vessels of the healthy lung indicating expression in vascular smooth muscle cells (arrowheads in Fig. 1F). In addition, SMC in bronchi were found to express NO-GC in previous studies [11]. Alveolar epithelial cells (AEC; identified by positive immunostaining using an antibody against thyroid transcription factor-1; TTF-1) expressed none of the pericyte markers (desmin, PDGFRβ, NG2, CD146) and, thus, are negative for NO-GC (see Additional file 1: Fig. S1K–N). These notions are corroborated by murine scRNAseq data (Fig. 1G, H) from two different sources (http://betsholtzlab.org/VascularSingleCells/database.html; [14], and www.LungEndothelialCellAtlas.com; [15]) and also hold true for the human lung (www.LungEndothelialCellAtlas.com). Although the single cell sequencing data show that PDGFRβ and NO-GC individually do not unambiguously identify pericytes and SMC, the combination of these two markers provides a much higher degree of specificity. Consequently, the combination of both signals allows to discriminate pericytes in lung parenchyma and SMC in blood vessels and airways.

### Bleomycin-induced pulmonary fibrosis

To investigate the fate of NO-GC-expressing cells during pulmonary fibrosis, we employed the bleomycin model [16, 17]. Bleomycin (2 U/kg) was administered via orotracheal instillation to WT mice at d0 and lungs were harvested at d21 (Additional file 1: Fig. S2A). Lung injury is evident by a reduction of anti-RAGE (receptor for advanced glycation endproducts)-immunolabeled alveolar epithelial cells type I and a strong increase in PDGFRβ immunoreactivity (Additional file 1: Fig. S2B). In addition, the signal intensity of PDGFRβ^+^ cells in fibrotic areas was higher than that in healthy lung tissue indicating elevated PDGFRβ expression. Cell number and cell density increased as judged by DAPI staining and DIC/pol images (Additional file 1: Fig. S2C). Dry lung weight as well as overall collagen expression increased in bleomycin-treated lungs (Additional file 1: Fig. S2D). Massive αSMA de novo expression was detected after bleomycin treatment indicating the formation of myofibroblasts, whereas αSMA signals were scarce in the untreated WT lung (Additional file 1: Fig. S2E). As shown in Additional file 1: Fig. S2e3, the few round fluorescent signals found in untreated WT lung in the absence of primary antibody probably arise from macrophage autofluorescence (see Additional file 1: Fig. S2e1). Incubation with secondary antibodies alone demonstrates antibody specificity (see Additional file 1: Fig. S2F). Together, these findings confirm the development of pulmonary fibrosis after bleomycin instillation.

### NO-GC differentiates two types of αSMA^+^ myofibroblasts in fibrotic lung

**Fig. 2:**
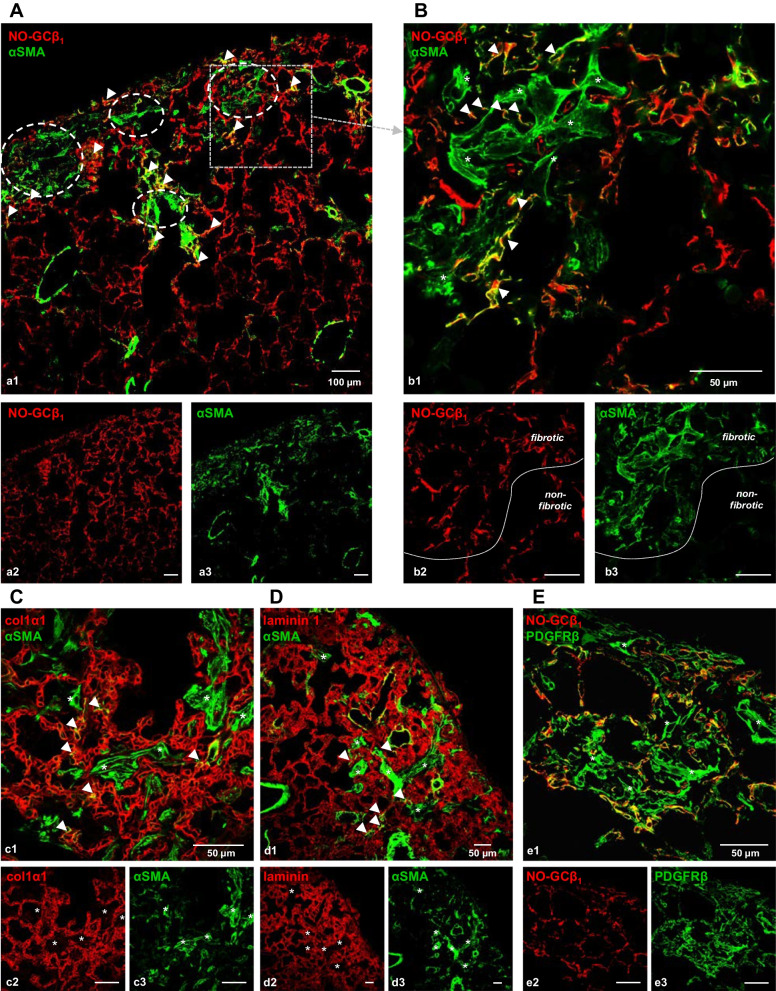
NO-GC expression distinguishes two types of myofibroblasts in fibrotic lung. Bleomycin was used to induce lung injury in WT mice. Lungs were isolated 21 days after bleomycin treatment. Lung tissue was stained with antibodies against NO-GCβ_1_, αSMA, col1α1, laminin 1 and PDGFRβ. **A** Overview of bleomycin-treated lung in which fibrotic areas can be discerned from non-fibrotic areas by parenchymal/extravascular αSMA expression (circled areas). **B** NO-GC expression distinguishes two types of αSMA + myofibroblasts (NO-GC^+^, yellow; arrowheads or NO-GC^−^, green; asterisks). Myofibroblasts are only found in the fibrotic area marked by the dotted lines in **b2**/**b3**. See Additional file 1: Fig. S2 for enlargement. **C**, **D** αSMA^+^ myofibroblasts (green) in the former lumen of alveoli defined by col1α1 signals (red) as marker of the alveolar wall or laminin 1 as marker of the basement membrane. Interstitial myofibroblasts are indicated by arrowheads. **E** PDGFRβ (green) co-localizes with NO-GC in pericytes and myofibroblasts in the alveolar wall (yellow). Intra-alveolar PDGFRβ^+^ myofibroblasts (asterisks in **d1**) do not stain for NO-GC. DAPI was used to stain nuclei (blue). Single channels are shown in **a2**–**e2** and **a3**–**e3**

Next, NO-GC expression after bleomycin instillation was evaluated. In fibrotic lung, similar to healthy lung, NO-GC is detectable in pericytes; also, NO-GC was found in vascular smooth muscle cells where it co-localized with αSMA (Fig. 2A; compare Fig. 1F). In addition, in the fibrotic area, a novel NO-GC/αSMA co-expressing cell turned up which was not located in airway or vessel walls but rather in the alveolar wall (arrowheads in fibrotic area of Fig. 2A).

Closer examination revealed that these NO-GC/αSMA co-expressing cells surrounded areas filled with a different type of αSMA-expressing cell that was negative for NO-GC (dotted circles in Fig. 2A, enlargement in Fig. 2B). These latter cells had a large multipolar appearance and intense αSMA-staining. As both cells show de novo αSMA expression, we define them as two different types of myofibroblasts based on differential NO-GC expression. The NO-GC^−^ myofibroblasts appeared to fill the former alveolar space (‘intra-alveolar’) and were surrounded by NO-GC^+^ myofibroblasts (see enlargement in Additional file 1: Fig. S3A). Closer analysis revealed that NO-GC^+^ myofibroblasts were indeed triple-positive for NO-GC/αSMA/PDGFRβ and in close contact with the intra-alveolar NO-GC^−^ myofibroblasts (Additional file 1: Fig. S3B–D).

Co-staining of αSMA and collagen 1 (col1α1), which labels the alveolar wall ([18]; see Additional file 1: Fig. S4B) or laminin 1, which marks the basement membrane ([19]; see Additional file 1: Fig. S4C), corroborated the intra-alveolar localization (in former alveoli) of these large NO-GC^−^ myofibroblasts (asterisks in Fig. 2C and D, see Additional file 1: Fig. S4D, E). Interestingly, the NO-GC^−^ myofibroblasts appear to expand over several alveoli (see Fig. 2B–D, Additional file 1: Fig. S3 and Additional file 1: Fig. S4D, E). In contrast, NO-GC^+^ myofibroblasts are embedded in the collagen matrix of the alveolar wall (arrowheads in Fig. 2C and D, see Additional file 1: Fig. S4D, E). Based on the differential localization, we will use the terms intra-alveolar and interstitial myofibroblasts.

Staining of PDGFRβ supported the differentiation into two distinct cell types (Fig. 2E): Based on immunofluorescence (see Fig. 1C) and RNAseq data (see Fig. 1G and H), cells co-expressing PDGFRβ and NO-GC (yellow) can be addressed as pericytes or SMC. As El Agha et al. have already shown that pre-existing SMC are not significantly amplified following bleomycin induction and, in addition, are not precursors of myofibroblasts [20], these interstitial myofibroblasts are likely to derive from pericytes. In addition, we also found PDGFRβ^+^ cells that were negative for NO-GC. According to their appearance as well as localization, they can be addressed as intra-alveolar myofibroblasts (asterisks in Fig. 2e1). Based on PDGFRβ expression, intra-alveolar myofibroblasts derive either from pericytes that lost NO-GC expression in the course of bleomycin injury, or, alternatively, from a non-pericytic progenitor cell that acquired de novo-PDGFRβ expression.

### Lineage tracing using tamoxifen-inducible PDGFRβ-CreER^T2^ mice

In order to solve this question and to determine the contribution of pericytes to the myofibroblast population, we established lineage tracing using a reporter mouse expressing the fluorescent dye tdTomato under control of the PDGFRβ promotor (PDGFRβ-CreER^T2^; R26tdTomato, hereafter abbreviated PDGFRβ-tdTomato mice). In contrast to previous publications using a constitutive PDGFRβ-Cre [21, 22], we here employed an inducible PDGFRβ-CreER^T2^ to prevent constitutive PDGFRβ-mediated cell labelling before or during the course of the experiment. Using this approach, determination of pericytes as precursors for the two different types of myofibroblast should be feasible.

Tamoxifen induction (without bleomycin at d0; treatment scheme in Additional file 1: Fig. S5A) led to PDGFRβ-CreER^T2^-dependent expression of tdTomato (Additional file 1: Fig. S5B–E). As expected, all tdTomato-expressing cells were immunopositive for PDGFRβ (co-expression 100% ± 0%; n = 102 PDGFRβ-tdTomato^+^ cells; Additional file 1: Fig. S5D) indicating the PDGFRβ promoter to be constitutive. In line with the co-immunostaining of NO-GC and PDGFRβ shown in Fig. 1C, NO-GC and PDGFRβ-tdTomato were found to be co-expressed (99.15% ± 0.85%; n = 105 PDGFRβ-tdTomato^+^ cells; Additional file 1: Fig. S5B; individual cells in C and E). Taken together, PDGFRβ-CreER^T2^-dependent tdTomato expression allows effective tracing of lung pericytes.

### Pericytes are precursors of interstitial but not intra-alveolar myofibroblasts

**Fig. 3:**
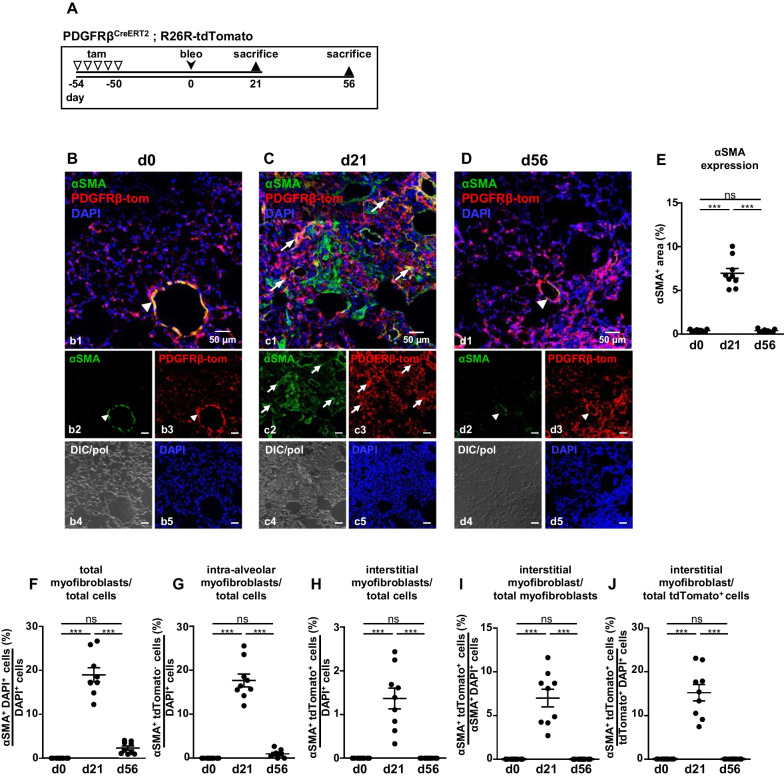
Myofibroblasts during lung fibrosis and fibrosis resolution. **A** Schematic illustration of the experimental setup for the induction of lung fibrosis. PDGFRβ-tdTomato reporter mice were injected with tamoxifen (tam) on 5 consecutive days to induce the expression of the fluorescent dye tdTomato under control of the PDGFRβ promotor (PDGFRβ-tom). 50 days later, mice were either sacrificed (= d0; **B**) or treated with bleomycin (bleo) and sacrificed after 21 (= d21; **C**) or 56 (= d56; **D**) days. Tissues show αSMA immunostaining (green) and PDGFRβ-tdTomato fluorescence (red). Arrows in (**C**) indicate myofibroblasts co-expressing αSMA and PDGFRβ-tdTomato. In (**B** and **D**), arrowheads indicate bronchial or vascular smooth muscle cells. DIC/pol illustrates tissue structure. DAPI was used to stain nuclei (blue). **E**–**J** Quantitative analyses of αSMA expression (20 × images), total myofibroblast proportion as well as proportions of intra-alveolar and interstitial myofibroblasts (63 × images, see Additional file 1: Fig. S5) for the respective time-points (n = 9 images from N = 3 animals; ns, not significant; *** = p < 0.001; one-way ANOVA followed by Tukey post-hoc test). Single channels are shown in **b2**–**d2**, **b3**–**d3**, **b4**–**d4** and **b5**–**d5**

To determine if pericytes transform to myofibroblasts and, if so, which of the two identified myofibroblast subtypes derives from this cell, we next investigated αSMA production in lungs from reporter mice that underwent the induction scheme shown in Fig. 3A. This scheme is based on the fact that the fibrotic response after bleomycin treatment is known to peak after approx. 21 days, and to be reversible with partial reconstitution of alveolar anatomy after > 50 days [23, 24]. To exclude the possibility of residual tamoxifen-induced recombination in cells that acquired PDGFRβ expression following injury or during disease progression, animals were kept for additional 50 days after the last tamoxifen injection before the administration of bleomycin (= d0).

In contrast to untreated lung (d0; Fig. 3B), which showed αSMA immunosignals only in bronchial and vascular smooth muscle cells, strong αSMA expression was detected in lung parenchyma at d21 after orotracheal bleomycin instillation (Fig. 3C); in fact, αSMA expression was identified in PDGFRβ-tdTomato^+^ cells as well as in cells lacking PDGFRβ-tdTomato. As El Agha et al. have already ruled out SMC as precursors of myofibroblasts [20], co-expression of αSMA and PDGFRβ-tdTomato indicates these cells to derive from pericytes (arrows in Fig. 3C); however, although very low in number, we cannot rule out PDGFRβ-expressing fibroblasts to also play a role. In contrast, shape and alveolar localization of PDGFRβ-tdTomato^−^ αSMA-expressing cells indicate identity with the intra-alveolar myofibroblasts described in Fig. 2. Lack of tdTomato expression in these cells suggests origin from a non-pericytic progenitor. Expectedly, αSMA expression vanished in the course of recovery up to d56 (compare Fig. 3C and D). The kinetics of αSMA expression are quantitatively analyzed in Fig. 3E.

In order to determine the relative proportion of the two different myofibroblasts, we analyzed 63 × images of lung tissue devoid of SMC in blood vessels or bronchi (Additional file 1: Fig. S6). The relative myofibroblast content increased up to almost 20% of all lung cells at d21 and declined to approx. 2% during recovery (Fig. 3F). Intra-alveolar myofibroblasts represented a frequent cell type (approx. 18% of all cells; Fig. 3G), whereas the interstitial, pericyte-derived myofibroblast was rare (approx. 1.5% of all cells; Fig. 3H). The majority of myofibroblasts were of the intra-alveolar type, whereas the interstitial, pericyte-derived myofibroblast made up only approx. 7% of total myofibroblasts and approx. 15% of all PDGFRβ-tdTomato^+^ cells (i.e., pericytes as SMC were excluded; Fig. 3I, J). Interestingly, interstitial myofibroblasts were not detectable at d56, whereas a small number of intra-alveolar cells could still be identified in 63 × images (unfilled arrowheads in Additional file 1: Fig. S6D).

### PDGFRβ-positive cells reduce NO-GC expression during the fibrotic reaction

**Fig. 4:**
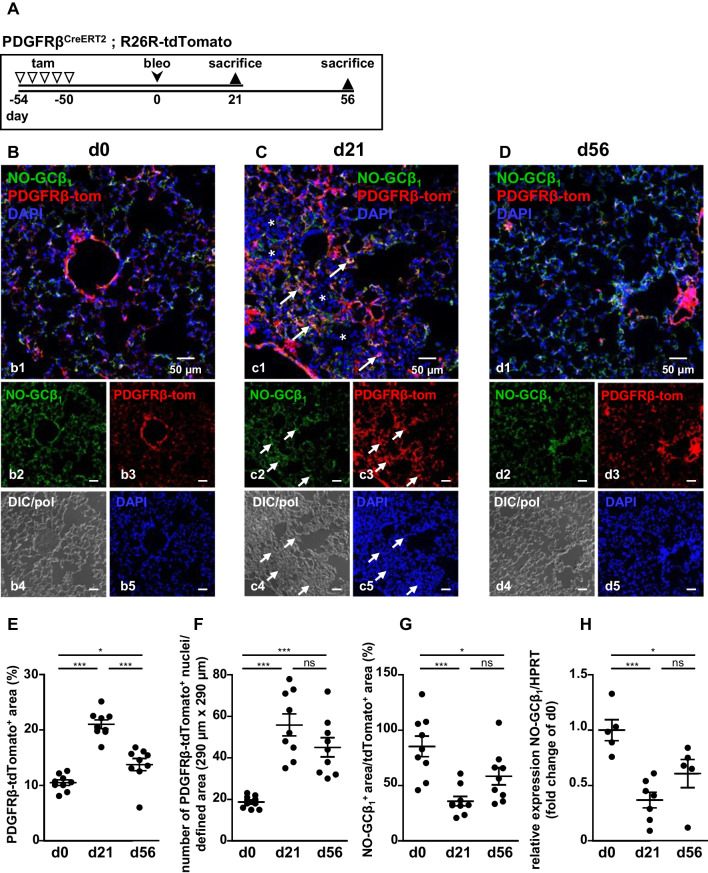
NO-GC expression during lung fibrosis and fibrosis resolution. **A** Schematic illustration of the experimental setup for the induction of lung fibrosis. PDGFRβ-tdTomato reporter mice were injected with tamoxifen (tam) on 5 consecutive days to induce the expression of the fluorescent dye tdTomato under control of the PDGFRβ promotor (PDGFRβ-tom). 50 days later, mice were either sacrificed (= d0; **B**) or treated with bleomycin (bleo) and sacrificed after 21 (= d21; **C**) or 56 (= d56; **D**) days. In (**C**), arrows indicate interstitial cells co-expressing NO-GC (green) and PDGFRβ-tdTomato (red) whereas asterisks indicate alveoli filled with NO-GC^−^/PDGFRβ-tdTomato^−^ cells. DIC/pol illustrates tissue structure. DAPI was used to stain nuclei (blue). **E–H** Quantitative analyses of PDGFRβ-tdTomato expression, number of PDGFRβ-tdTomato^+^ nuclei, NO-GCβ_1_/PDGFRβ-tdTomato expression (n = 9 images from N = 3 animals, 63 × images, see Additional file 1: Fig. S6) and relative expression of NO-GCβ_1_/HPRT using qPCR (d0: N = 5 animals; d21: n = 7 animals; d56: N = 5 animals; ns, not significant; * = p < 0.05; ** = p < 0.01; *** = p < 0.001; one-way ANOVA followed by Tukey post-hoc test). Single channels are shown in **b2**–**d2**, **b3**–**d3**, **b4**–**d4** and **b5**–**d5**

To study NO-GC expression during the fibrotic reaction (d21) and during fibrosis resolution (d56), we treated PDGFRβ-tdTomato reporter mice according to the scheme in Fig. 4A. As pre-existing SMC do not significantly amplify following bleomycin treatment [20], PDGFRβ-tdTomato-expressing cells (mostly pericytes) can be shown to strongly increase in number within the first 21 days of the bleomycin response, followed by a slight reversal during the recovery phase (Fig. 4b3–d3 and Additional file 1: Fig. S7; quantified as PDGFRβ-tdTomato^+^ area in Fig. 4E and PDGFRβ-tdTomato^+^ nuclei in Fig. 4F). NO-GC was detectable in pericytes and SMC under control conditions, during fibrosis as well as during resolution (Fig. 4B–D). PDGFRβ-tdTomato^+^ cells retain NO-GC expression (Additional file 1: Fig. S7), albeit strongly reduced during fibrosis (indicated by the ratio of NO-GCβ_1_-to-PDGFRβ-tdTomato immunosignals; Fig. 4G). qPCR corroborated this suppressed expression of NO-GC (Fig. 4H). In addition, cells expressing NO-GC de novo do not develop during fibrosis or fibrosis recovery (Additional file 1: Fig. S7). In summary, NO-GC is strongly down-regulated during the fibrotic response.

### Pericyte-derived interstitial myofibroblasts produce collagen type 1

**Fig. 5:**
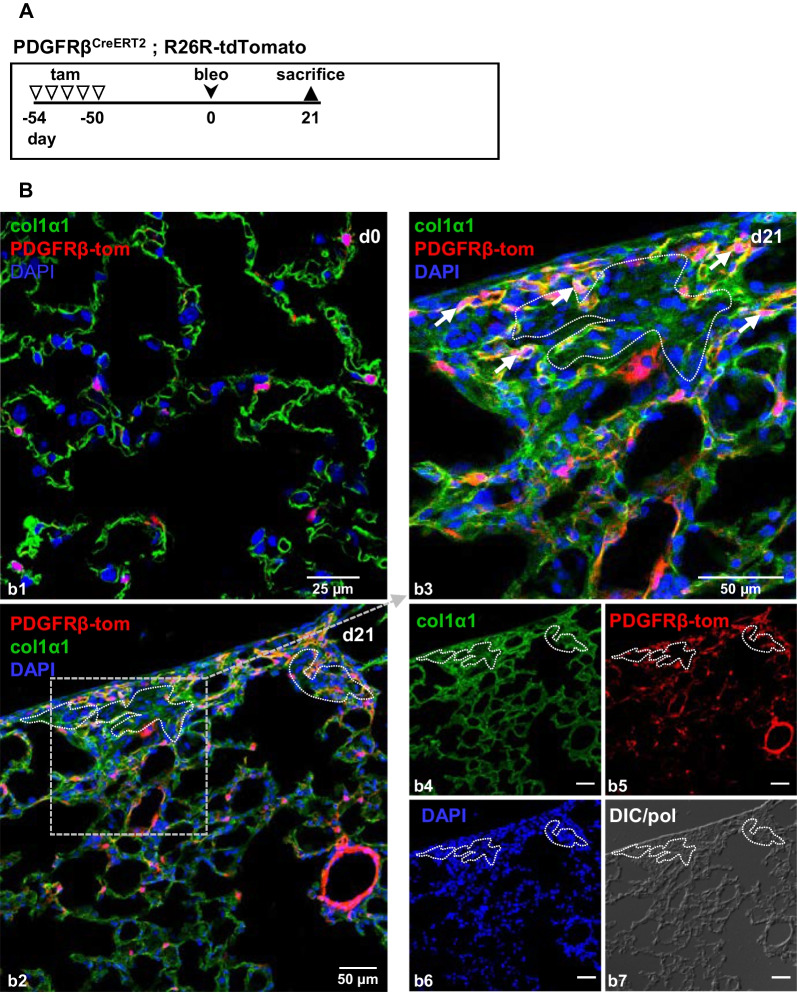
Pericyte-derived myofibroblasts produce collagen 1 after bleomycin injury. **A** Schematic illustration of the experimental setup for the induction of lung fibrosis. PDGFRβ-tdTomato reporter mice were injected with tamoxifen (tam) on 5 consecutive days to induce the expression of the fluorescent dye tdTomato under control of the PDGFRβ promotor (PDGFRβ-tom). 50 days later, mice were either sacrificed (= d0) or treated with bleomycin (bleo) and sacrificed after 21 (= d21). **B** PDGFRβ-tdTomato^+^ lungs (red) were co-stained with an antibody against col1α1 (green). In healthy lung tissue (d0), PDGFRβ-tdTomato^+^ cells are localized in the alveolar wall indicated by col1a1 staining (**b1**). In fibrotic lung, co-staining of PDGFRβ-tdTomato and col1α1 (yellow) indicates ECM production by pericyte-derived myofibroblasts of the alveolar wall. Overview is shown in **b2**, single channels are shown in **b4**–**b7**. Fibrotic areas of indicated areas are enlarged in **b3**. Dotted lines indicate areas filled with intra-alveolar myofibroblasts (note their nuclei in **b6** and cellular structure in **b7**). DAPI was used to stain nuclei (blue). DIC/pol illustrates tissue structure

Using col1α1-GFP mice, recent studies have shown that PDFGRβ^+^ cells do not express collagen 1 under healthy conditions [23] and only a minority of αSMA^+^ myofibroblasts produce collagen 1 in the course of bleomycin-induced lung fibrosis [21]. Therefore, we intended to clarify whether NO-GC^+^ pericyte-derived myofibroblasts of the alveolar wall engage in the production of extracellular matrix. Lungs from untreated (d0) and 21 days after bleomycin-treated PDGFRβ-tdTomato mice were isolated (Fig. 5A) and probed with an antibody against col1α1. In healthy lung tissue, PDGFRβ-tdTomato^+^ cells are localized in the alveolar wall defined by col1a1 staining, but do not show production of collagen 1 (Fig. 5b1). In contrast, Fig. 5b2 shows a fibrotic area (enlarged in Fig. 5b3) characterized by increased cell occurrence (as shown with DAPI and DIC/pol), elevated PDGFRβ-tdTomato fluorescence as well as more intense staining for col1α1 (Fig. 5b4–b7). Co-localization of PDGFRβ-tdTomato and col1α1 indicates strong ECM production by the rare pericyte-derived interstitial myofibroblasts (Fig. 5b3, arrows), which is reminiscent of the low number of col1α1-GFP^+^ myofibroblasts shown in [21]. Former alveolar areas (identified by high DAPI and DIC/pol signals; dotted lines; Fig. 5b2–7) showed diffuse col1α1 staining. Based on the fact that only little association between col1α1 and αSMA expression has been shown [21, 23], we are currently unable to differentiate whether the faint col1a1 stems from interstitial or intra-alveolar myofibroblasts.

## Discussion

### NO-GC is a marker of PDGFRβ-positive lung cells

Although the high expression of NO-GC in lung tissue has long been known and exploited for purification [24, 25], the specific cell type expressing the enzyme has only recently been characterized [11]. Using immunofluorescence, we identified PDGFRβ^+^ cells as the main NO-GC-expressing cell type in murine lung. Based on the morphology of these cells with prominent nuclei and several long processes the majority of these cells are likely to be pericytes in healthy lung. This assumption is corroborated by the fact that PDGFRβ is expressed constitutively on lung pericytes [26]. Besides these parenchymal cells, we also detected NO-GC in SMC.

There is no single specific marker for pericytes, and several proteins have been shown to label these cells in an organ- and development-specific expression pattern [9]. Here, we show co-expression of four of the most established pericyte markers, i.e., desmin, PDGFRβ, NG2, and CD146 with NO-GC. The single cell sequencing data (Fig. 1G and H) do not show absolute specificity of PDGFRβ or NO-GC for pericytes and SMC when used individually. However, the combination of these two markers allows for a high degree of specificity. The morphohistological and immunofluorescent findings in combination with scRNA expression data favor these NO-GC^+^/PDGFRβ^+^/PDGFRβ-dtTomato^+^ cells as (mostly) pericytes.

Using the inducible PDGFRβ-CreER^T2^ strain in combination with a tdTomato reporter mouse line, we were therefore able to lineage trace NO-GC-expressing pericytes and SMC during the fibrotic response. As El Agha et al. have already shown that pre-existing SMC neither significantly amplify following bleomycin treatment nor contribute to the myofibroblast pool [20], our data show that pericytes are precursors for a specific type of myofibroblast that can be localized in the alveolar wall (see below). During pericyte-to-myofibroblast transformation, the cellular NO-GC expression is adapted: Despite an increase in NO-GC-expressing cells, net NO-GC expression (mRNA and protein) is reduced (but not lost) in lineage traced cells. In addition, we did not see development of de novo NO-GC-expressing cells (i.e., without co-expressing tdTomato) during fibrosis. Taken together, these data allow us to use NO-GC as novel pericyte-specific marker for further experiments. The exact function of NO/cGMP signaling in pericytes/myofibroblasts remains to be established even though a role in wound healing and fibrosis appears likely. We expect NO-GC to be involved in the regulation of extracellular matrix deposition and myofibroblast contractility.

### Role of PDGFRβ-positive cells as myofibroblast precursors

PDGFRβ-expressing mesenchymal cells contribute to the fibrotic reaction in various organs after injury. This cell population has been shown to mediate fibrotic responses in liver, kidney, heart, skeletal muscle and lung [27–31]. Accordingly, constitutive and tamoxifen-inducible PDGFRβ-Cre models have been exploited to study the role of PDGFRβ-expressing cells not only regarding the mechanisms of fibrosis [22] but also to describe the role of pericytes in angiogenesis [32] and pericyte-to-myofibroblast transition after knee surgery [33].

### Origin and markers of interstitial and intra-alveolar myofibroblasts

We here identify two types of myofibroblasts that can be differentiated 1. by their expression of NO-GC, 2. by their localization in injured lung tissue, 3. by their shape and 4. by their lineage (Table 2). Both myofibroblast types have in common that they develop exclusively after injury showing de novo αSMA expression. Interestingly, only NO-GC as marker allowed the differentiation of the two myofibroblasts whereas expression of PDGFRβ, the commonly used marker for pericytes, did not permit this distinction based on the de novo PDGFRβ synthesis by intra-alveolar myofibroblasts (Additional file 1: Fig. S8).

**Table 2:** Characteristics of myofibroblast subtypes

	Interstitial myofibroblast	Intra-alveolar myofibroblast
Localisation	Interstitial	Intra-alveolar
NO-GC	Positive	Negative
Precursor	Pericytes	Not determined
Shape	Pericyte-like	Multipolar; spread over several alveoli
PDGFRβ expression	Constitutive	de novo after fibrotic insult
αSMA expression	de novo	de novo
Collagen type 1 synthesis	de novo	Not determined
Possible functions	- Angiogenesis - Blood flow regulation - Generation of collagen and tensile force - Mechanotransduction - Immune modulation	- Alveolar spaceholder - Generation of extracellular matrix and tensile force - Re-epithelialization

The first type of myofibroblast expresses NO-GC, localizes in the alveolar wall, derives from pericytes as shown by lineage tracing using a PDGFRβ-tdTomato reporter strain and produces collagen type 1. The second type of myofibroblast is negative for NO-GC, does not derive from PDGFRβ^+^ pericytes (even though it expresses PDGFRβ de novo after injury), has a large multipolar shape and appears to fill and spread over alveolar spaces within the injured areas.

The classification of pericyte-derived and non-pericyte-derived myofibroblasts (PDGFRβ-constitutively-expressing and PDGFRβ*-*de novo-expressing, respectively) indicates different roles of pulmonary myofibroblasts. With PDGFRβ as a 'specific' marker for pericytes, PDGFRβ-regulated Cre expression has been used to trace the fate of these cells. However, the constitutive PDGFRβ-Cre line employed in other studies [21, 22] does not allow discriminating between the two types of myofibroblasts identified in this study, as, after injury, both indistinguishably express PDGFRβ. Here, we employed the tamoxifen inducible PDGFRβ-CreER^T2^ to induce temporally controlled labeling of cells with active PDGFRβ promotor in the adult lung; in fact, as the intra-alveolar myofibroblasts were found to acquire PDGFRβ expression after injury, only the inducible lineage tracing strategy allowed us to differentiate between the two types of myofibroblasts. Using a PDGFRβ-tdTomato reporter mouse in combination with the fact that SMC do not contribute to myofibroblasts [20], the interstitial myofibroblast can be shown to derive from NO-GC^+^/PDGFRβ^+^ pericytes. We have not yet determined the precursor for the intra-alveolar myofibroblast, although we can exclude pericytes based on our lineage tracing studies. Lipofibroblasts have been shown to be potential myofibroblasts precursors [20]; whether this cell type is linked to the formation of intra-alveolar myofibroblasts has yet to be clarified.

Intriguingly, using single-cell RNA sequencing, Xie et al. [34] have recently shown a newly emerging type of mesenchymal cell after bleomycin treatment, which is characterized by high PDGFRβ expression in combination with expression of both NO-GC isoforms as well as αSMA. The authors denote these cells as ‘PDGFRβ hi’ fibroblasts; however, based on the absence of a uniquely defined pericyte cluster in their analysis, the authors do not explicitly rule out this novel mesenchymal cell to be pericyte-derived. As in our study, the combination of PDGFRβ, NO-GC as well as αSMA expression only occurred in pericyte-derived myofibroblasts after injury, we judge our interstitial cells and the new ‘PDGFRβ hi’ fibroblast type to be identical. This assumption is supported by the fact that the ‘PDGFRβ hi’ fibroblast is a rare cell type (approx. 1.3% of all mesenchymal cells), as is our pericyte-derived myofibroblast (approx. 1.5% of all cells). In addition, our second, NO-GC^−^ myofibroblast might correspond to the main type of myofibroblast identified in that study characterized by high αSMA expression. Thus, our data confirms the RNA data by Xie et al. [34] and, in addition, identifies not only the precursors of the ‘PDGFRβ hi’ fibroblasts as pericytes but also the exact localization of both myofibroblast types in fibrotic lung tissue.

## Conclusions

Taken together, we show that two different myofibroblast types can be differentiated on the basis of NO-GC expression. We have not yet elucidated the exact function of NO-GC in the small, but seemingly important pool of myofibroblasts that evolve under fibrotic conditions. Conceivably, the known anti-fibrotic mechanism of NO-GC includes the modulation of collagen type 1 deposition. Myofibroblasts have been suggested as potential targets for the therapy of pulmonary fibrosis [35, 36]. In fact, RNAseq analyses of lung tissue from IPF patients identify a cell type which is triple positive for NO-GC/αSMA/PDGFRβ (http://www.ipfcellatlas.com; [37]; Additional file 1: Fig. S9). This cell type is conceivable to be the human IPF counterpart to the pericyte-derived, NO-GC^+^, interstitial myofibroblast in this study. Our data, however, indicate that myofibroblasts should probably not be addressed as a homogeneous target cell type within this disease.

## Supplementary Information

**Additional file 1. Fig. S1:** Evaluation of NO-GC-expressing cell types in murine lung. **Fig S2:** Bleomycin-induced lung injury in WT mice. **Fig. S3:** Two types of myofibroblasts can be differentiated by NO-GC expression and extra/intra-alveolar localization. **Fig. S4:** Control stainings of control and bleomycin-treated lung tissue. **Fig. S5:** Lineage tracing of PDGFR β-positive cells in murine lung. **Fig. S6:** Lineage tracing of pericyte-derived myofibroblasts. **Fig. S7:** NO-GC is expressed in PDGFRβ-tomato^+^ cells at all stages of the fibrotic response. **Fig. S8:** Intra-alveolar myofibroblasts express PDGFRβ denovo. **Fig. S9:** RNAseq data of lung tissue from IPF patients.

## Data Availability

The datasets used and/or analyzed during the current study are available from the corresponding author on reasonable request.

## Abbreviations

αSMA: α Smooth muscle actin
col1α1: Collagen type 1α1
ECM: Extracellular matrix
NG2: Neural/glial antigen 2
NO-GC: NO-sensitive guanylyl cyclase
NO-GCβ_1_: β_1_ Subunit of NO-sensitive guanylyl cyclase
PDGFRβ: Platelet-derived growth factor β
SMC: Smooth muscle cell
TTF-1: Thyroid transcription factor-1
