# Supplementary material for: NO-sensitive guanylyl cyclase discriminates pericyte-derived interstitial from intra-alveolar myofibroblasts in murine pulmonary fibrosis
Source: Respir Res. 2023 Jun 22;24:167. doi: 10.1186/s12931-023-02479-2 (PMC10288756; doi:10.1186/s12931-023-02479-2)
Supplement: Supplementary file 1 — Additional file 1. Fig. S1: Evaluation of NO-GC-expressing cell types in murine lung. Fig S2: Bleomycin-induced lung injury in WT mice. Fig. S3: Two types of myofibroblasts can be differentiated by NO-GC expression and extra/intra-alveolar localization. Fig. S4: Control stainings of control and bleomycin-treated lung tissue. Fig. S5: Lineage tracing of PDGFR β-positive cells in murine lung. Fig. S6: Lineage tracing of pericyte-derived myofibroblasts. Fig. S7: NO-GC is expressed in PDGFRβ-tomato+ cells at all stages of the fibrotic response. Fig. S8: Intra-alveolar myofibroblasts express PDGFRβ denovo. Fig. S9: RNAseq data of lung tissue from IPF patients. [file 12931_2023_2479_MOESM1_ESM.pptx]

## Slide 1
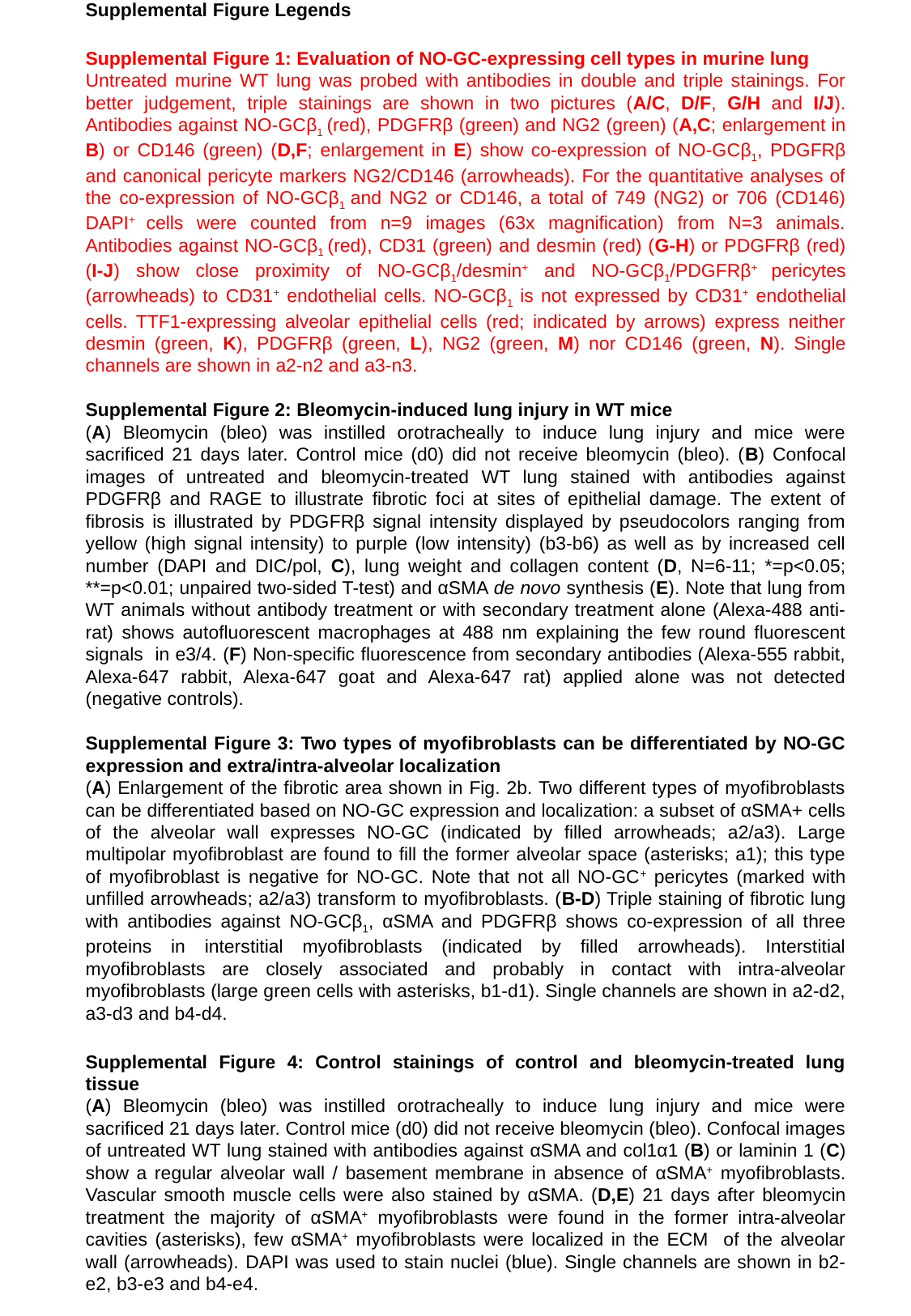

Supplemental Figure Legends
Supplemental Figure 1: Evaluation of NO-GC-expressing cell types in murine lung
Untreated murine WT lung was probed with antibodies in double and triple stainings. For better judgement, triple stainings are shown in two pictures (A/C, D/F, G/H and I/J). Antibodies against NO-GCβ1 (red), PDGFRβ (green) and NG2 (green) (A,C; enlargement in B) or CD146 (green) (D,F; enlargement in E) show co-expression of NO-GCβ1, PDGFRβ and canonical pericyte markers NG2/CD146 (arrowheads). For the quantitative analyses of the co-expression of NO-GCβ1 and NG2 or CD146, a total of 749 (NG2) or 706 (CD146) DAPI+ cells were counted from n=9 images (63x magnification) from N=3 animals. Antibodies against NO-GCβ1 (red), CD31 (green) and desmin (red) (G-H) or PDGFRβ (red) (I-J) show close proximity of NO-GCβ1/desmin+ and NO-GCβ1/PDGFRβ+ pericytes (arrowheads) to CD31+ endothelial cells. NO-GCβ1 is not expressed by CD31+ endothelial cells. TTF1-expressing alveolar epithelial cells (red; indicated by arrows) express neither desmin (green, K), PDGFRβ (green, L), NG2 (green, M) nor CD146 (green, N). Single channels are shown in a2-n2 and a3-n3.
Supplemental Figure 2: Bleomycin-induced lung injury in WT mice
(A) Bleomycin (bleo) was instilled orotracheally to induce lung injury and mice were sacrificed 21 days later. Control mice (d0) did not receive bleomycin (bleo). (B) Confocal images of untreated and bleomycin-treated WT lung stained with antibodies against PDGFRβ and RAGE to illustrate fibrotic foci at sites of epithelial damage. The extent of fibrosis is illustrated by PDGFRβ signal intensity displayed by pseudocolors ranging from yellow (high signal intensity) to purple (low intensity) (b3-b6) as well as by increased cell number (DAPI and DIC/pol, C), lung weight and collagen content (D, N=6-11; *=p<0.05; **=p<0.01; unpaired two-sided T-test) and αSMA de novo synthesis (E). Note that lung from WT animals without antibody treatment or with secondary treatment alone (Alexa-488 anti-rat) shows autofluorescent macrophages at 488 nm explaining the few round fluorescent signals in e3/4. (F) Non-specific fluorescence from secondary antibodies (Alexa-555 rabbit, Alexa-647 rabbit, Alexa-647 goat and Alexa-647 rat) applied alone was not detected (negative controls).
Supplemental Figure 3: Two types of myofibroblasts can be differentiated by NO-GC expression and extra/intra-alveolar localization
(A) Enlargement of the fibrotic area shown in Fig. 2b. Two different types of myofibroblasts can be differentiated based on NO-GC expression and localization: a subset of αSMA+ cells of the alveolar wall expresses NO-GC (indicated by filled arrowheads; a2/a3). Large multipolar myofibroblast are found to fill the former alveolar space (asterisks; a1); this type of myofibroblast is negative for NO-GC. Note that not all NO-GC+ pericytes (marked with unfilled arrowheads; a2/a3) transform to myofibroblasts. (B-D) Triple staining of fibrotic lung with antibodies against NO-GCβ1, αSMA and PDGFRβ shows co-expression of all three proteins in interstitial myofibroblasts (indicated by filled arrowheads). Interstitial myofibroblasts are closely associated and probably in contact with intra-alveolar myofibroblasts (large green cells with asterisks, b1-d1). Single channels are shown in a2-d2, a3-d3 and b4-d4.
Supplemental Figure 4: Control stainings of control and bleomycin-treated lung tissue
(A) Bleomycin (bleo) was instilled orotracheally to induce lung injury and mice were sacrificed 21 days later. Control mice (d0) did not receive bleomycin (bleo). Confocal images of untreated WT lung stained with antibodies against αSMA and col1α1 (B) or laminin 1 (C) show a regular alveolar wall / basement membrane in absence of αSMA+ myofibroblasts. Vascular smooth muscle cells were also stained by αSMA. (D,E) 21 days after bleomycin treatment the majority of αSMA+ myofibroblasts were found in the former intra-alveolar cavities (asterisks), few αSMA+ myofibroblasts were localized in the ECM of the alveolar wall (arrowheads). DAPI was used to stain nuclei (blue). Single channels are shown in b2-e2, b3-e3 and b4-e4.

## Slide 2
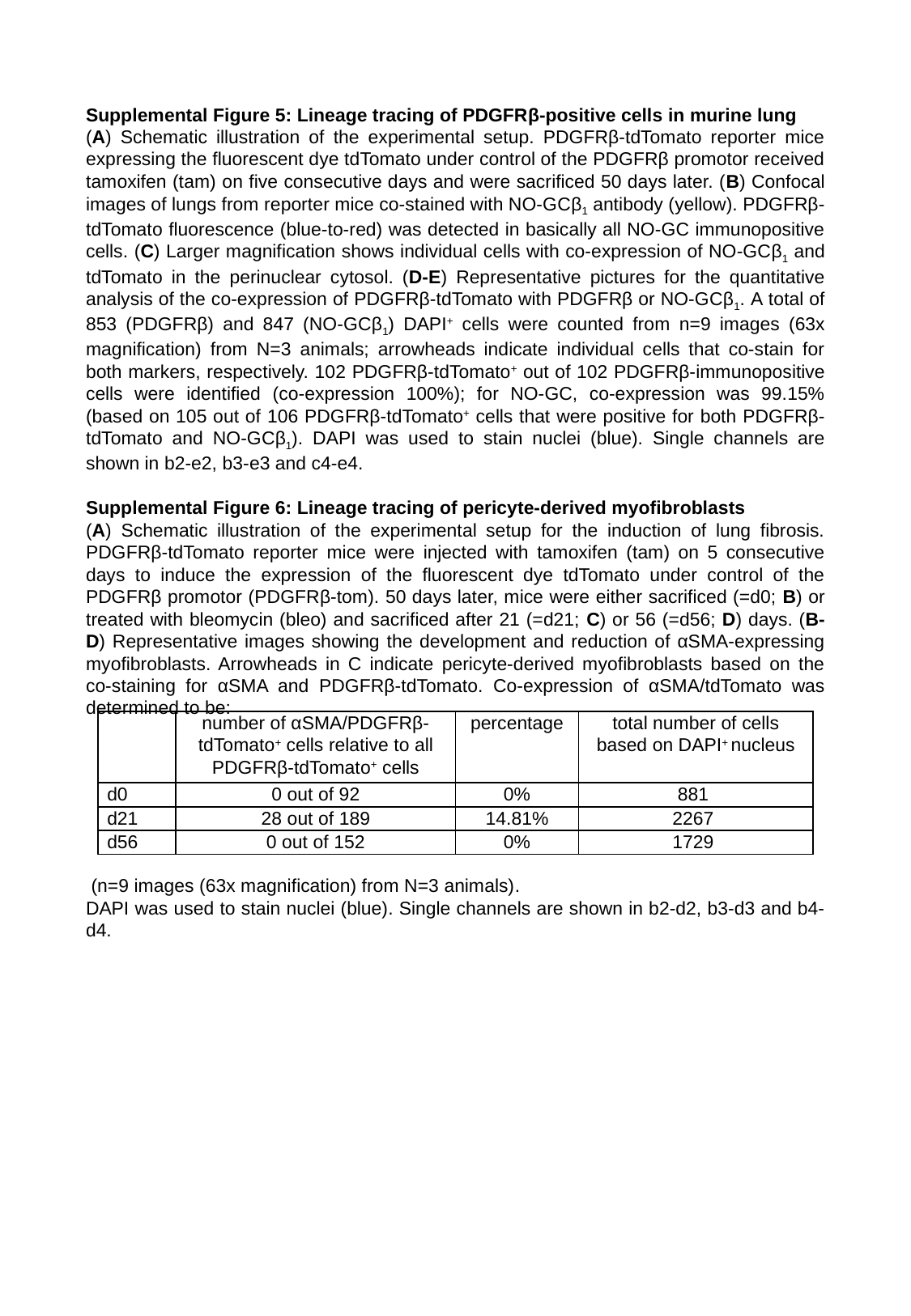

Supplemental Figure 5: Lineage tracing of PDGFRβ-positive cells in murine lung
(A) Schematic illustration of the experimental setup. PDGFRβ-tdTomato reporter mice expressing the fluorescent dye tdTomato under control of the PDGFRβ promotor received tamoxifen (tam) on five consecutive days and were sacrificed 50 days later. (B) Confocal images of lungs from reporter mice co-stained with NO-GCβ1 antibody (yellow). PDGFRβ-tdTomato fluorescence (blue-to-red) was detected in basically all NO-GC immunopositive cells. (C) Larger magnification shows individual cells with co-expression of NO-GCβ1 and tdTomato in the perinuclear cytosol. (D-E) Representative pictures for the quantitative analysis of the co-expression of PDGFRβ-tdTomato with PDGFRβ or NO-GCβ1. A total of 853 (PDGFRβ) and 847 (NO-GCβ1) DAPI+ cells were counted from n=9 images (63x magnification) from N=3 animals; arrowheads indicate individual cells that co-stain for both markers, respectively. 102 PDGFRβ-tdTomato+ out of 102 PDGFRβ-immunopositive cells were identified (co-expression 100%); for NO-GC, co-expression was 99.15% (based on 105 out of 106 PDGFRβ-tdTomato+ cells that were positive for both PDGFRβ-tdTomato and NO-GCβ1). DAPI was used to stain nuclei (blue). Single channels are shown in b2-e2, b3-e3 and c4-e4.
Supplemental Figure 6: Lineage tracing of pericyte-derived myofibroblasts
(A) Schematic illustration of the experimental setup for the induction of lung fibrosis. PDGFRβ-tdTomato reporter mice were injected with tamoxifen (tam) on 5 consecutive days to induce the expression of the fluorescent dye tdTomato under control of the PDGFRβ promotor (PDGFRβ-tom). 50 days later, mice were either sacrificed (=d0; B) or treated with bleomycin (bleo) and sacrificed after 21 (=d21; C) or 56 (=d56; D) days. (B-D) Representative images showing the development and reduction of αSMA-expressing myofibroblasts. Arrowheads in C indicate pericyte-derived myofibroblasts based on the co-staining for αSMA and PDGFRβ-tdTomato. Co-expression of αSMA/tdTomato was determined to be:
 (n=9 images (63x magnification) from N=3 animals).
DAPI was used to stain nuclei (blue). Single channels are shown in b2-d2, b3-d3 and b4-d4.
| | number of αSMA/PDGFRβ-tdTomato+ cells relative to all PDGFRβ-tdTomato+ cells | percentage | total number of cells based on DAPI+ nucleus |
| --- | --- | --- | --- |
| d0 | 0 out of 92 | 0% | 881 |
| d21 | 28 out of 189 | 14.81% | 2267 |
| d56 | 0 out of 152 | 0% | 1729 |

## Slide 3
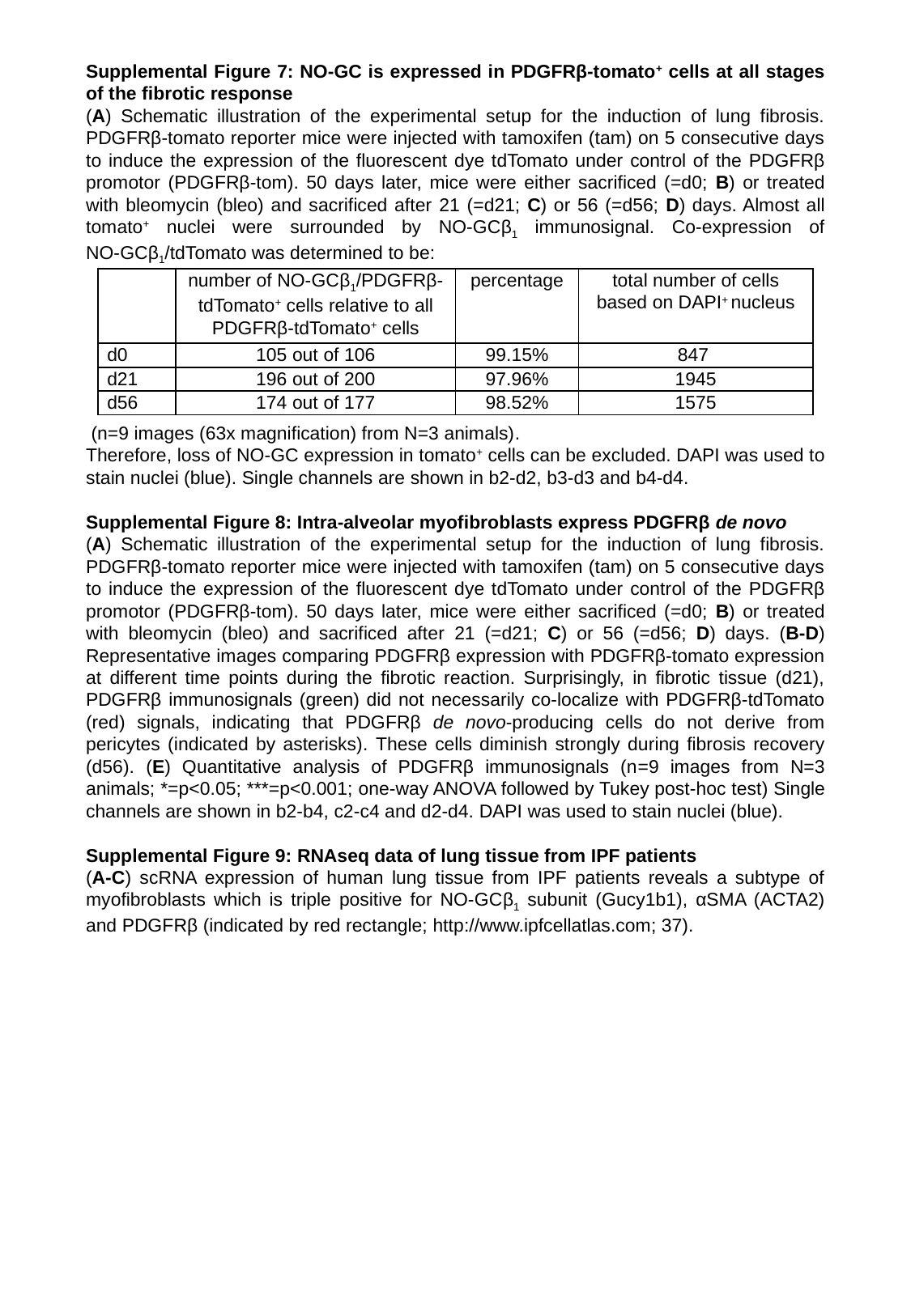

Supplemental Figure 7: NO-GC is expressed in PDGFRβ-tomato+ cells at all stages of the fibrotic response
(A) Schematic illustration of the experimental setup for the induction of lung fibrosis. PDGFRβ-tomato reporter mice were injected with tamoxifen (tam) on 5 consecutive days to induce the expression of the fluorescent dye tdTomato under control of the PDGFRβ promotor (PDGFRβ-tom). 50 days later, mice were either sacrificed (=d0; B) or treated with bleomycin (bleo) and sacrificed after 21 (=d21; C) or 56 (=d56; D) days. Almost all tomato+ nuclei were surrounded by NO-GCβ1 immunosignal. Co-expression of NO-GCβ1/tdTomato was determined to be:
 (n=9 images (63x magnification) from N=3 animals).
Therefore, loss of NO-GC expression in tomato+ cells can be excluded. DAPI was used to stain nuclei (blue). Single channels are shown in b2-d2, b3-d3 and b4-d4.
Supplemental Figure 8: Intra-alveolar myofibroblasts express PDGFRβ de novo
(A) Schematic illustration of the experimental setup for the induction of lung fibrosis. PDGFRβ-tomato reporter mice were injected with tamoxifen (tam) on 5 consecutive days to induce the expression of the fluorescent dye tdTomato under control of the PDGFRβ promotor (PDGFRβ-tom). 50 days later, mice were either sacrificed (=d0; B) or treated with bleomycin (bleo) and sacrificed after 21 (=d21; C) or 56 (=d56; D) days. (B-D) Representative images comparing PDGFRβ expression with PDGFRβ-tomato expression at different time points during the fibrotic reaction. Surprisingly, in fibrotic tissue (d21), PDGFRβ immunosignals (green) did not necessarily co-localize with PDGFRβ-tdTomato (red) signals, indicating that PDGFRβ de novo-producing cells do not derive from pericytes (indicated by asterisks). These cells diminish strongly during fibrosis recovery (d56). (E) Quantitative analysis of PDGFRβ immunosignals (n=9 images from N=3 animals; *=p<0.05; ***=p<0.001; one-way ANOVA followed by Tukey post-hoc test) Single channels are shown in b2-b4, c2-c4 and d2-d4. DAPI was used to stain nuclei (blue).
Supplemental Figure 9: RNAseq data of lung tissue from IPF patients
(A-C) scRNA expression of human lung tissue from IPF patients reveals a subtype of myofibroblasts which is triple positive for NO-GCβ1 subunit (Gucy1b1), αSMA (ACTA2) and PDGFRβ (indicated by red rectangle; http://www.ipfcellatlas.com; 37).
| | number of NO-GCβ1/PDGFRβ-tdTomato+ cells relative to all PDGFRβ-tdTomato+ cells | percentage | total number of cells based on DAPI+ nucleus |
| --- | --- | --- | --- |
| d0 | 105 out of 106 | 99.15% | 847 |
| d21 | 196 out of 200 | 97.96% | 1945 |
| d56 | 174 out of 177 | 98.52% | 1575 |

## Slide 4
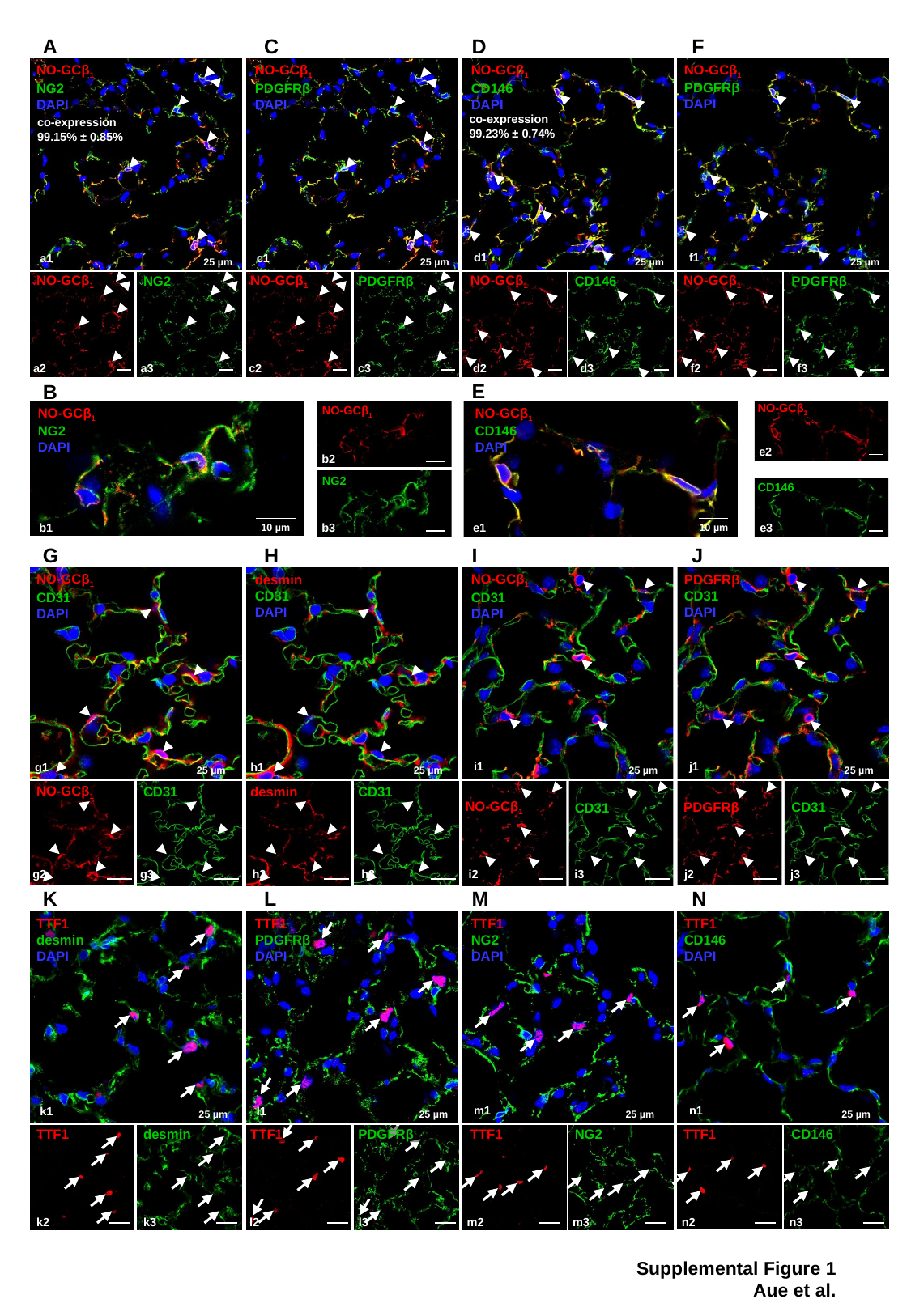

A
C
D
F
NO-GCβ1
PDGFRβ
DAPI
NO-GCβ1
NG2
DAPI
NO-GCβ1
PDGFRβ
DAPI
NO-GCβ1
CD146
DAPI
co-expression
99.23% ± 0.74%
co-expression
99.15% ± 0.85%
f1
d1
a1
c1
25 µm
25 µm
25 µm
25 µm
NO-GCβ1
NG2
NO-GCβ1
PDGFRβ
NO-GCβ1
CD146
NO-GCβ1
PDGFRβ
d2
d3
f2
f3
a2
a3
c2
c3
E
B
NO-GCβ1
NO-GCβ1
NG2
DAPI
NO-GCβ1
CD146
DAPI
NO-GCβ1
e2
b2
NG2
CD146
e1
b1
b3
e3
10 µm
10 µm
G
H
I
J
PDGFRβ
CD31
DAPI
NO-GCβ1
CD31
DAPI
desmin
CD31
DAPI
NO-GCβ1
CD31
DAPI
j1
i1
g1
h1
25 µm
25 µm
25 µm
25 µm
NO-GCβ1
CD31
desmin
CD31
NO-GCβ1
PDGFRβ
CD31
CD31
g2
g3
h2
h3
i2
i3
j2
j3
K
L
M
N
TTF1
CD146
DAPI
TTF1
desmin
DAPI
TTF1
PDGFRβ
DAPI
TTF1
NG2
DAPI
n1
m1
k1
l1
25 µm
25 µm
25 µm
25 µm
TTF1
desmin
TTF1
PDGFRβ
TTF1
NG2
TTF1
CD146
k2
k3
l2
l3
m2
m3
n2
n3
Supplemental Figure 1
Aue et al.

## Slide 5
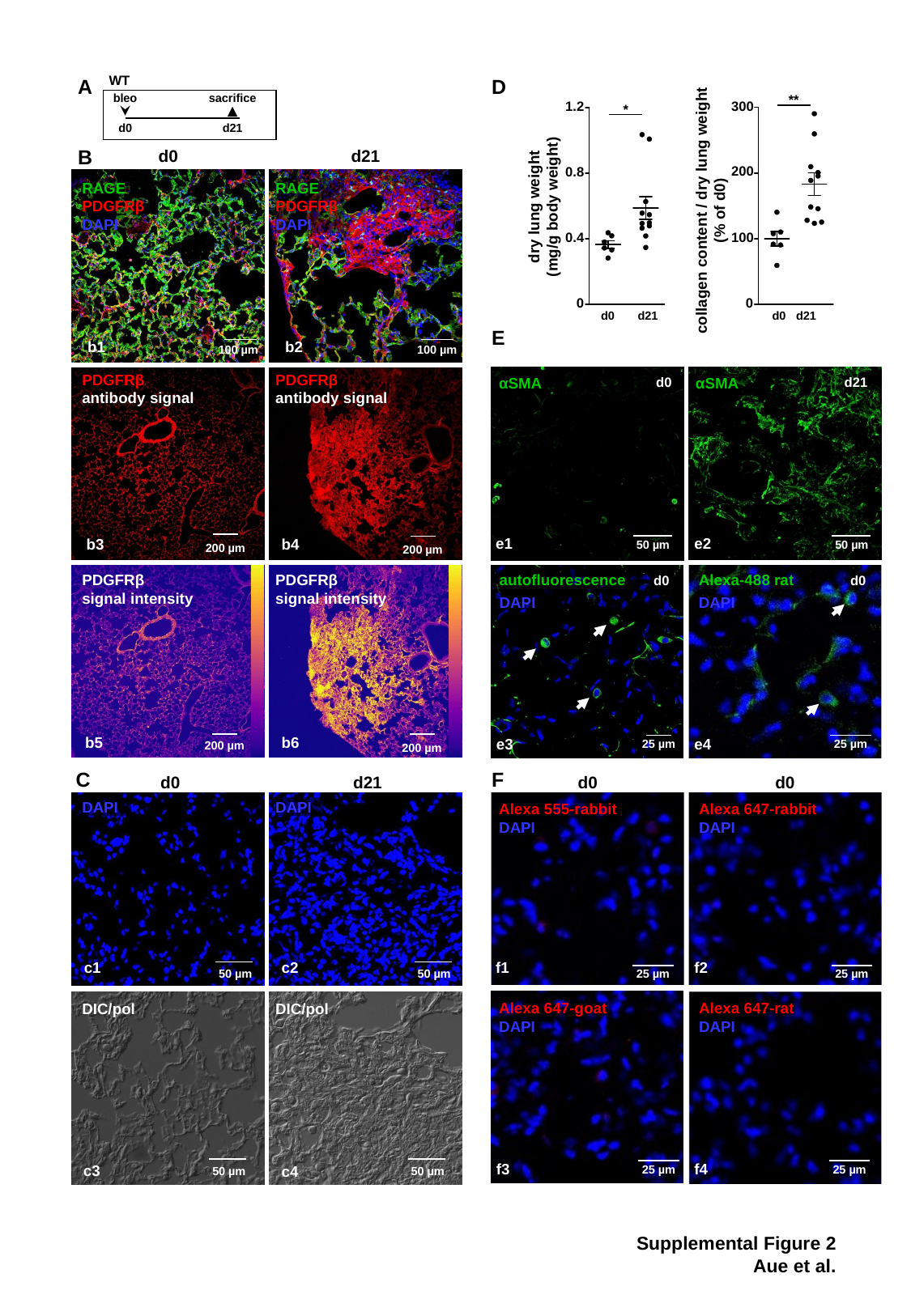

A
D
WT
bleo
sacrifice
**
1.2
300
*
d0
d21
B
d0
d21
200
0.8
RAGE
PDGFRβ
DAPI
RAGE
PDGFRβ
DAPI
dry lung weight
(mg/g body weight)
collagen content / dry lung weight
(% of d0)
100
0.4
0
0
d0
d21
d0
d21
E
b1
b2
100 µm
100 µm
PDGFRβ
antibody signal
PDGFRβ
antibody signal
αSMA
d0
αSMA
d21
e1
e2
b3
b4
50 µm
50 µm
200 µm
200 µm
autofluorescence
Alexa-488 rat
PDGFRβ
signal intensity
PDGFRβ
signal intensity
d0
d0
DAPI
DAPI
F
b5
b6
e3
e4
25 µm
25 µm
200 µm
200 µm
C
F
d0
d21
d0
d0
DAPI
DAPI
Alexa 555-rabbit
DAPI
Alexa 647-rabbit
DAPI
f1
f2
c1
c2
25 µm
25 µm
50 µm
50 µm
Alexa 647-goat
DAPI
Alexa 647-rat
DAPI
DIC/pol
DIC/pol
f3
f4
c3
c4
25 µm
25 µm
50 µm
50 µm
Supplemental Figure 2
Aue et al.

## Slide 6
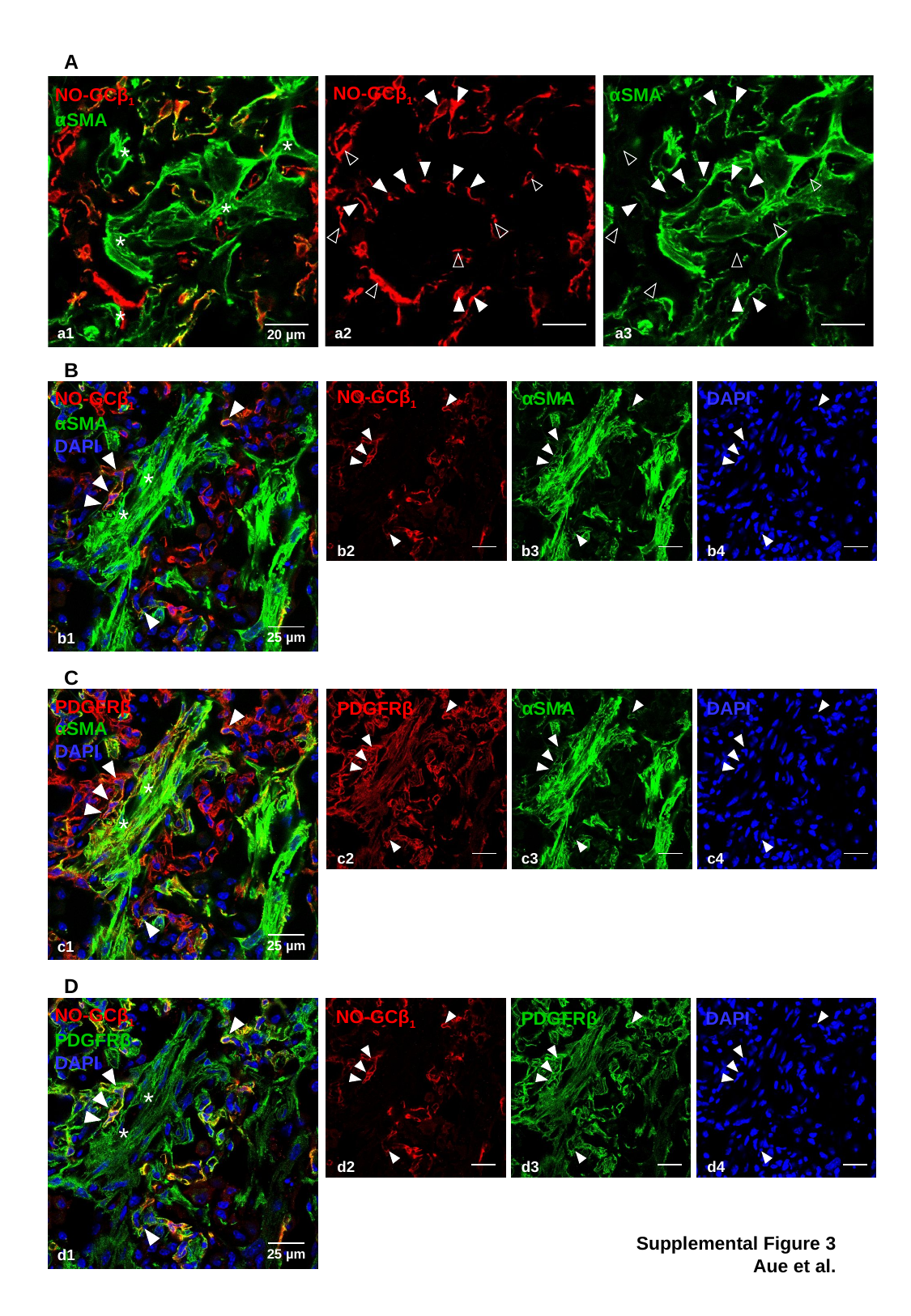

A
NO-GCβ1
αSMA
NO-GCβ1
αSMA
*
*
*
*
*
a1
a2
a3
20 µm
B
NO-GCβ1
αSMA
DAPI
NO-GCβ1
αSMA
DAPI
*
*
b2
b3
b4
b1
25 µm
C
PDGFRβ
αSMA
DAPI
PDGFRβ
αSMA
DAPI
*
*
c2
c3
c4
c1
25 µm
D
NO-GCβ1
PDGFRβ
DAPI
NO-GCβ1
PDGFRβ
DAPI
*
*
d2
d3
d4
Supplemental Figure 3
Aue et al.
25 µm
d1

## Slide 7
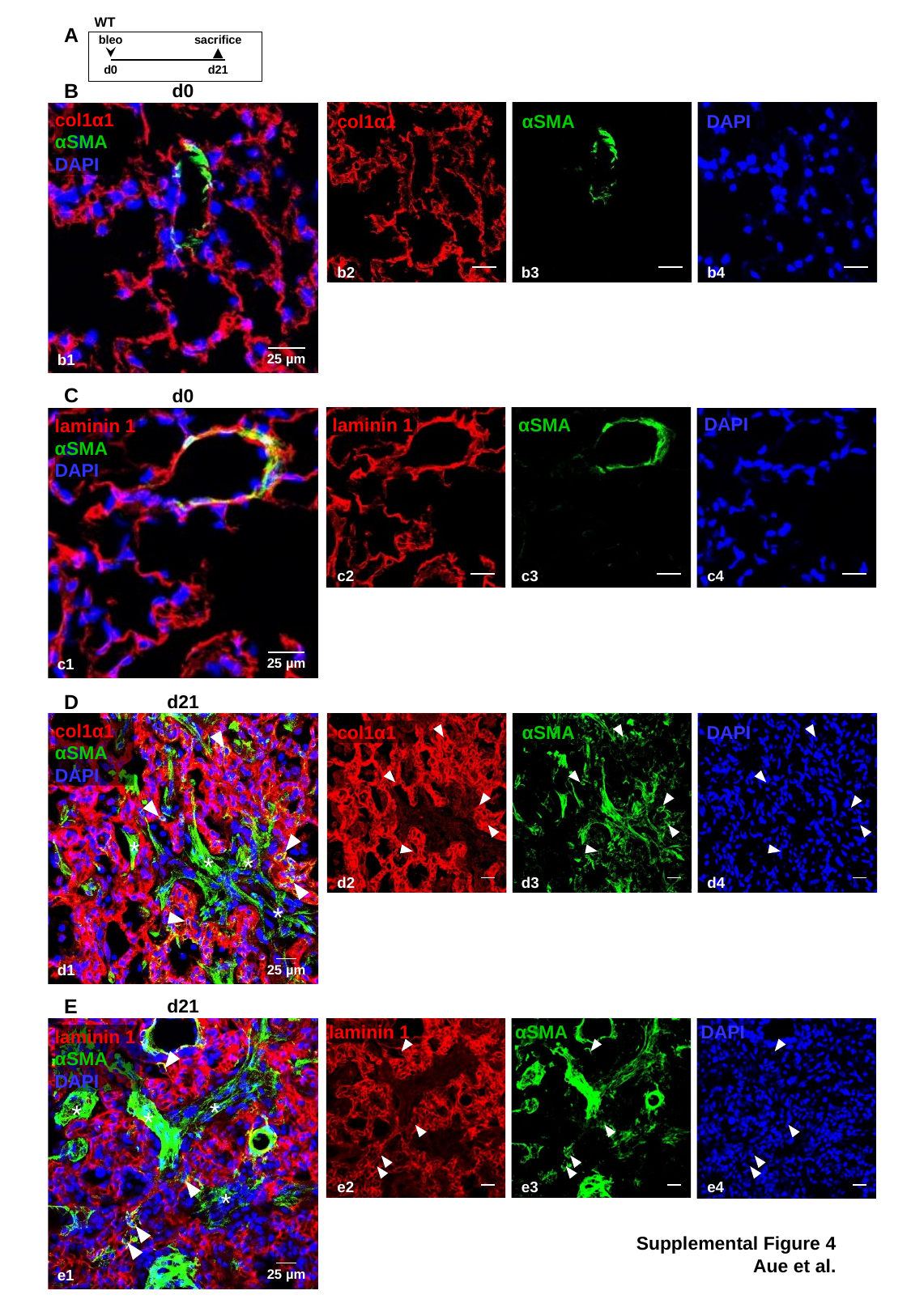

WT
A
bleo
sacrifice
d0
d21
B
d0
col1α1
αSMA
DAPI
col1α1
αSMA
DAPI
b2
b3
b4
b1
25 µm
C
d0
laminin 1
αSMA
DAPI
laminin 1
αSMA
DAPI
c2
c3
c4
25 µm
c1
D
d21
col1α1
αSMA
DAPI
col1α1
αSMA
DAPI
*
*
*
d2
d3
d4
*
d1
25 µm
E
d21
laminin 1
αSMA
DAPI
laminin 1
αSMA
DAPI
*
*
*
*
e2
e3
e4
Supplemental Figure 4
Aue et al.
25 µm
e1

## Slide 8
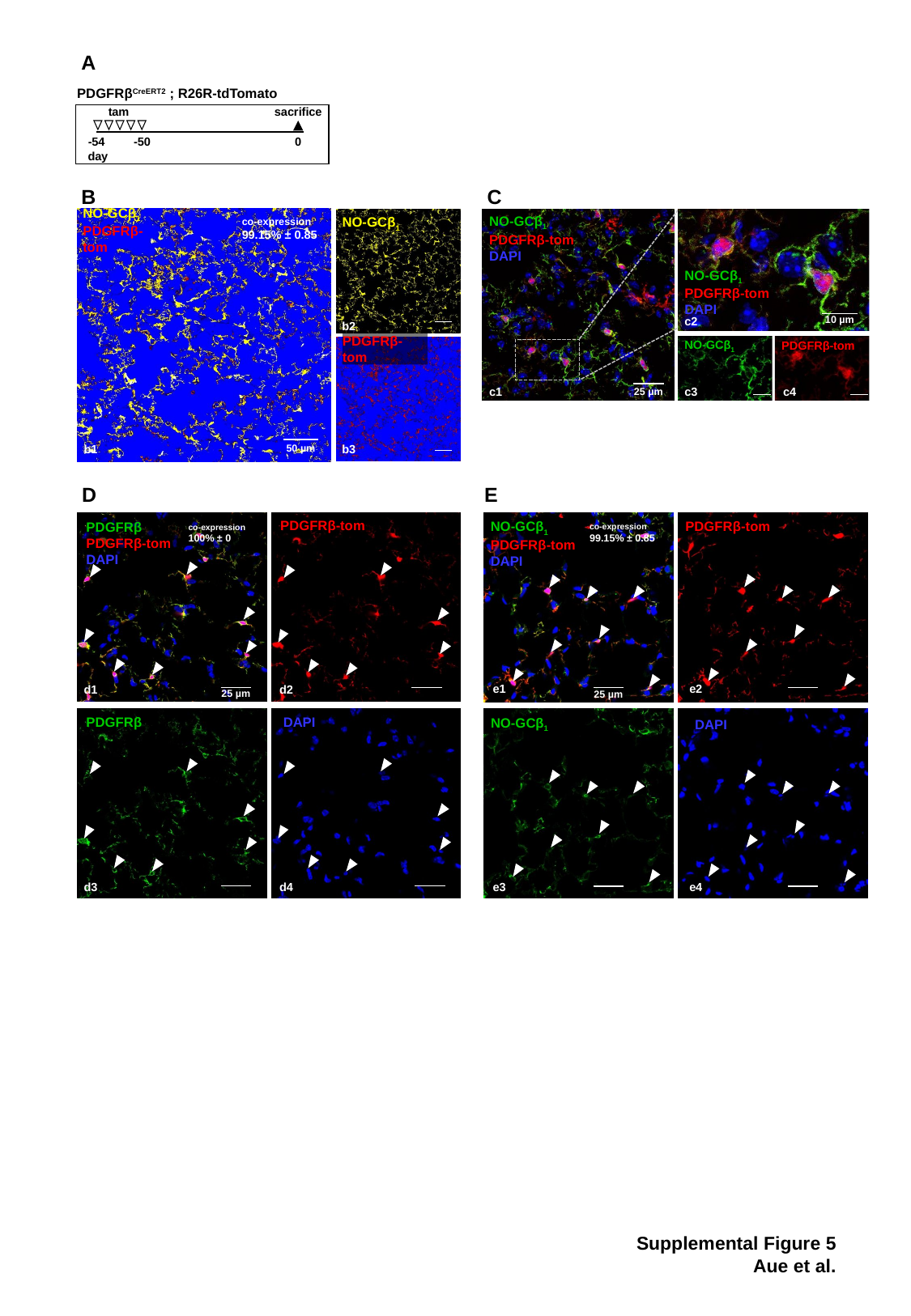

A
PDGFRβCreERT2 ; R26R-tdTomato
tam
sacrifice
-54
-50
0
day
B
C
25 µm
co-expression
99.15% ± 0.85
NO-GCβ1
PDGFRβ-tom
NO-GCβ1
PDGFRβ-tom
DAPI
NO-GCβ1
NO-GCβ1
PDGFRβ-tom
DAPI
10 µm
c2
b2
NO-GCβ1
PDGFRβ-tom
PDGFRβ-tom
c1
c3
c4
b1
b3
50 µm
D
E
co-expression
99.15% ± 0.85
co-expression
100% ± 0
PDGFRβ
PDGFRβ-tom
DAPI
PDGFRβ-tom
PDGFRβ-tom
NO-GCβ1
PDGFRβ-tom
DAPI
e1
e2
d1
d2
25 µm
25 µm
PDGFRβ
DAPI
DAPI
NO-GCβ1
d3
d4
e3
e4
Supplemental Figure 5
Aue et al.

## Slide 9
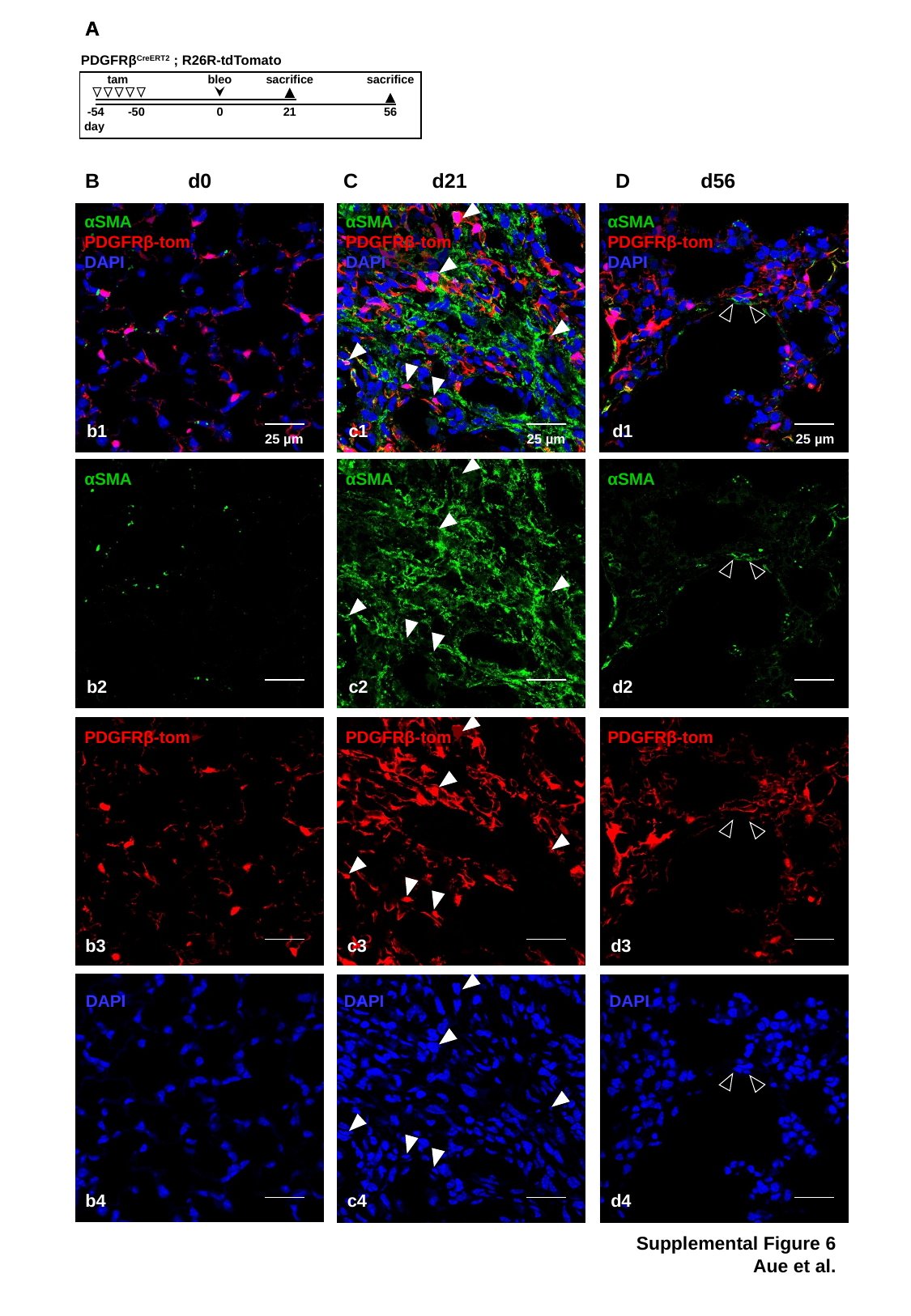

A
A
PDGFRβCreERT2 ; R26R-tdTomato
tam
bleo
sacrifice
sacrifice
-54
-50
0
21
56
day
B
d0
C
d21
D
d56
αSMA
PDGFRβ-tom
DAPI
αSMA
PDGFRβ-tom
DAPI
αSMA
PDGFRβ-tom
DAPI
b1
b2
b3
b4
c1
c2
c3
c4
d1
d2
d3
d4
25 µm
25 µm
25 µm
αSMA
αSMA
αSMA
PDGFRβ-tom
PDGFRβ-tom
PDGFRβ-tom
DAPI
DAPI
DAPI
Supplemental Figure 6
Aue et al.

## Slide 10
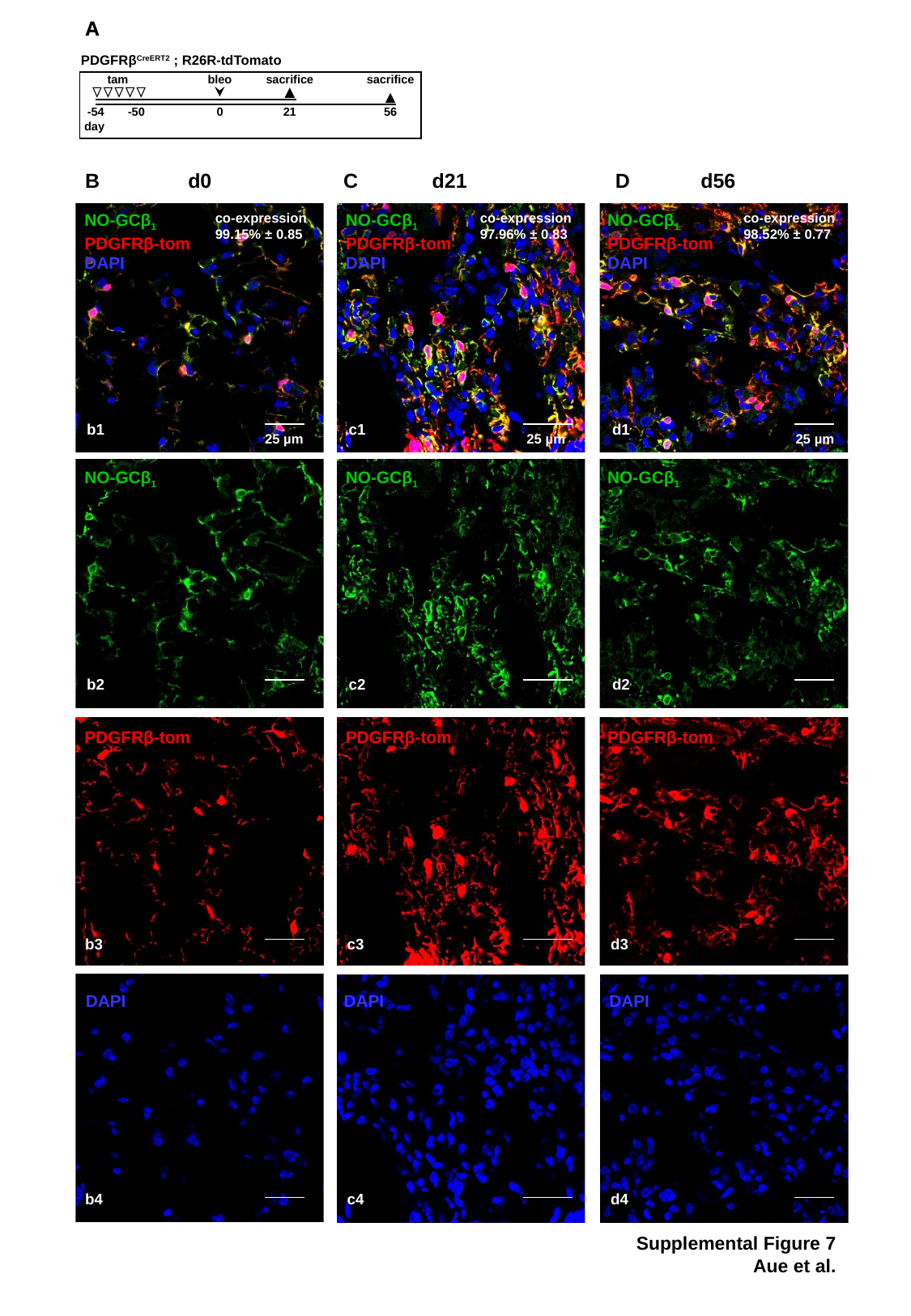

A
A
PDGFRβCreERT2 ; R26R-tdTomato
tam
bleo
sacrifice
sacrifice
-54
-50
0
21
56
day
B
d0
C
d21
D
d56
co-expression
99.15% ± 0.85
co-expression
97.96% ± 0.83
co-expression
98.52% ± 0.77
NO-GCβ1
PDGFRβ-tom
DAPI
NO-GCβ1
PDGFRβ-tom
DAPI
NO-GCβ1
PDGFRβ-tom
DAPI
b1
c1
d1
25 µm
25 µm
25 µm
NO-GCβ1
NO-GCβ1
NO-GCβ1
b2
c2
d2
PDGFRβ-tom
PDGFRβ-tom
PDGFRβ-tom
b3
c3
d3
DAPI
DAPI
DAPI
b4
c4
d4
Supplemental Figure 7
Aue et al.

## Slide 11
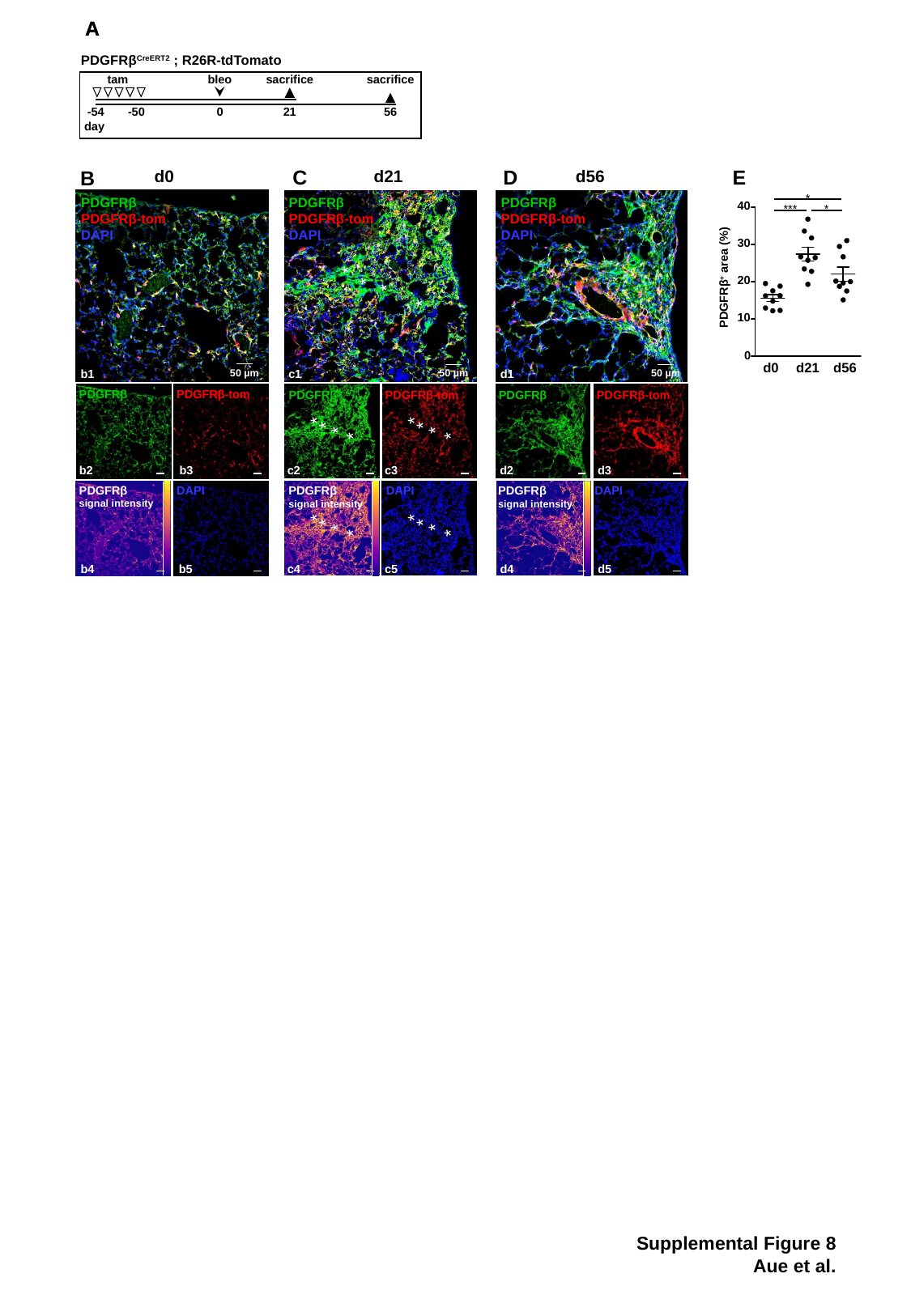

A
A
PDGFRβCreERT2 ; R26R-tdTomato
tam
bleo
sacrifice
sacrifice
-54
-50
0
21
56
day
D
E
C
B
d0
d21
d56
*
PDGFRβ
PDGFRβ-tom
DAPI
PDGFRβ
PDGFRβ-tom
DAPI
PDGFRβ
PDGFRβ-tom
DAPI
40
*
***
30
*
*
*
*
PDGFRβ+ area (%)
20
10
0
d0
d21
d56
50 µm
b1
c1
d1
50 µm
50 µm
PDGFRβ
PDGFRβ-tom
PDGFRβ
PDGFRβ-tom
PDGFRβ
PDGFRβ-tom
*
*
*
*
*
*
*
*
b2
b3
c2
c3
d2
d3
PDGFRβ
signal intensity
DAPI
PDGFRβ
signal intensity
DAPI
PDGFRβ
signal intensity
DAPI
*
*
*
*
*
*
*
*
b4
b5
c4
c5
d4
d5
Supplemental Figure 8
Aue et al.

## Slide 12
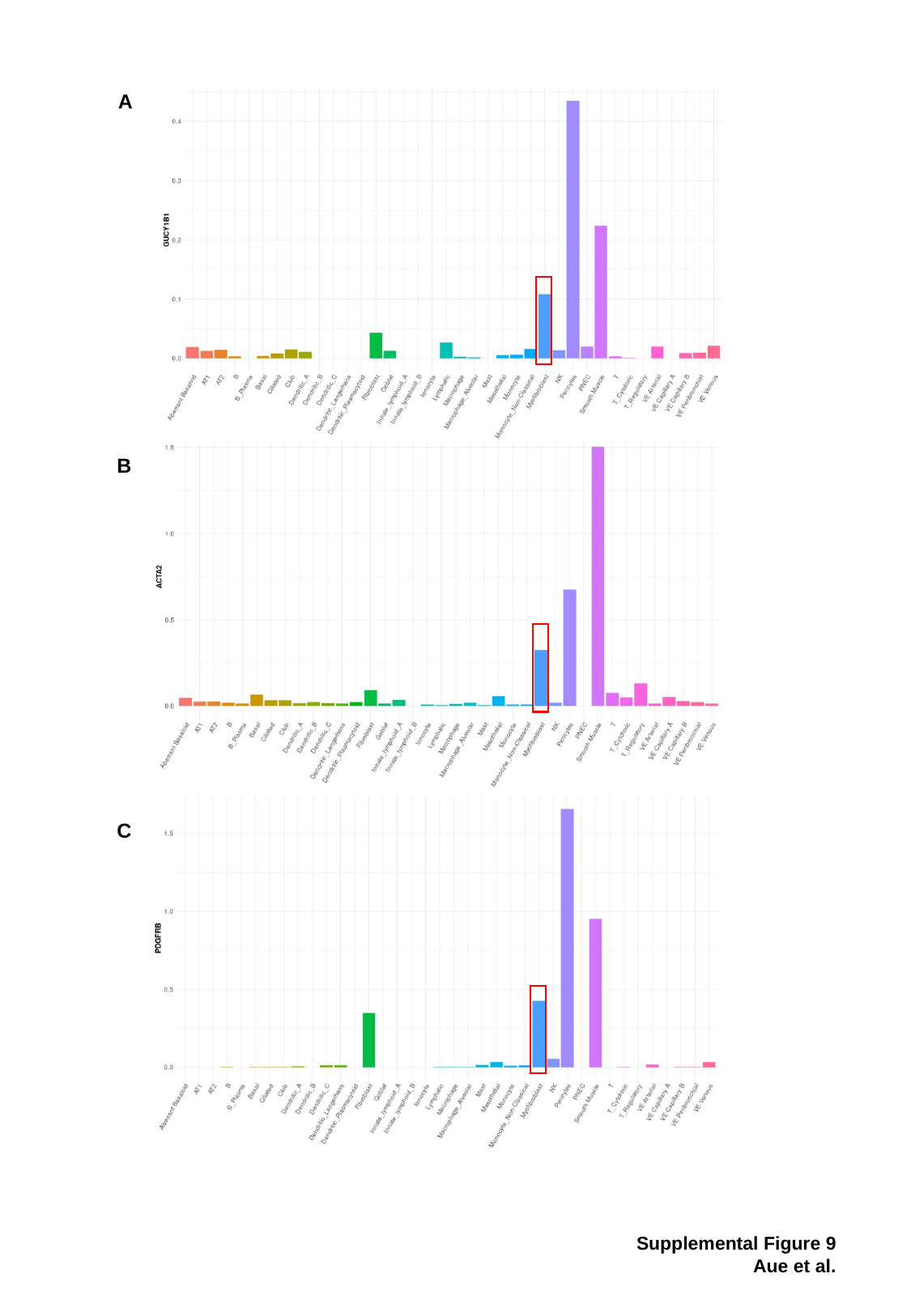

A
B
C
Supplemental Figure 9
Aue et al.
